# Visualization of renal rotenone accumulation after oral administration and *in situ* detection of kidney injury biomarkers via MALDI mass spectrometry imaging

**DOI:** 10.3389/fmolb.2024.1366278

**Published:** 2024-07-01

**Authors:** Chuckcris P. Tenebro, Neaven Bon Joy M. Marcial, Janine J. Salcepuedes, Josie C. Torrecampo, Rajelle D. Hernandez, John Alfon P. Francisco, Kristine Mae G. Infante, Veronica J. Belardo, Monissa C. Paderes, Rita Grace Y. Alvero, Jonel P. Saludes, Doralyn S. Dalisay

**Affiliations:** ^1^ Center for Chemical Biology and Biotechnology, University of San Agustin, Iloilo City, Philippines; ^2^ Institute of Chemistry, University of the Philippines Diliman, Quezon City, Philippines; ^3^ Pharmalytics Corporation, General Trias City, Cavite, Philippines; ^4^ Center for Natural Drug Discovery and Development, University of San Agustin, Iloilo City, Philippines; ^5^ Department of Chemistry, University of San Agustin, Iloilo City, Philippines; ^6^ Balik Scientist Program, Department of Science and Technology—Philippine Council for Health Research and Development, Taguig City, Philippines; ^7^ Department of Biology, University of San Agustin, Iloilo City, Philippines

**Keywords:** MALDI MSI, rotenone, kidney tissues, drug metabolism, toxicity, endogenous metabolite, anticancer

## Abstract

The examination of drug accumulation within complex biological systems offers valuable insights into the molecular aspects of drug metabolism and toxicity. Matrix-assisted laser desorption/ionization mass spectrometry imaging (MALDI MSI) is an innovative methodology that enables the spatial visualization and quantification of biomolecules as well as drug and its metabolites in complex biological system. Hence, this method provides valuable insights into the metabolic profile and any molecular changes that may occur as a result of drug treatment. The renal system is particularly vulnerable to adverse effects of drug-induced harm and toxicity. In this study, MALDI MSI was utilized to examine the spatial distribution of drug and renal metabolites within kidney tissues subsequent to a single oral dosage of the anticancer compound rotenone. The integration of ion mobility spectrometry with MALDI MSI enhanced the data acquisition and analysis, resulting to improved mass resolution. Subsequently, the MS/MS fragment ions of rotenone reference drug were detected and characterized using MALDI HDMS/MS imaging. Notably, drug accumulation was observed in the cortical region of the representative kidney tissue sections treated with rotenone. The histological examination of treated kidney tissues did not reveal any observable changes. Differential ion intensity of renal endogenous metabolites was observed between untreated and rotenone-treated tissues. In the context of treated kidney tissues, the ion intensity level of sphingomyelin (D18:1/16:0), a sphingolipid indicator of glomerular cell injury and renal damage, was found to be elevated significantly compared to untreated kidney tissues. Conversely, the ion intensities of choline, glycero-3-phosphocholine (GPC), inosine, and a lysophosphatidylcholine LysoPC(18:0) exhibited a significant decrease. The results of this study demonstrate the potential of MALDI MSI as a novel technique for investigating the *in situ* spatial distribution of drugs and renal endogenous molecules while preserving the anatomical integrity of the kidney tissue. This technique can be used to study drug-induced metabolism and toxicity in a dynamic manner.

## Introduction

Multiple strategies have been utilized in absorption, distribution, metabolism, and excretion (ADME) studies to examine the pharmacokinetic (PK) characteristics and possible safety issues associated with drug candidates (Vrbanac and Slauter, 2017). Following this, the incorporation of computational modeling (*in silico*), laboratory experiments (*in vitro*), and animal studies (*in vivo*) has significantly enhanced the evaluation of drugs’ ADME characteristics (Honório et al., 2013). Commonly employed techniques for the examination of specific metabolites in tissue samples include liquid/gas chromatography-mass spectrometry (LC/GC-MS) and ligand-binding assays. Wherein, it is necessary to perform tissue homogenization, which may potentially lead to reduced spatial resolution (Guo et al., 2022). The limitations inherent in traditional methodologies were effectively mitigated through the utilization of matrix-assisted laser desorption/ionization mass spectrometry imaging (MALDI MSI). This is a robust analytical technique that facilitates the identification, measurement, and depiction of drugs and naturally occurring compounds *in situ* within biological specimens. MALDI MSI minimizes the occurrence of sample degradation to maintain its original state (Spruill et al., 2022). MALDI MSI enhanced the spatial resolution of complex biological system when combined with ion mobility spectrometry (IMS), thereby enabling ion discrimination based on their structural dimensions and mass (Dodds and Baker, 2019).

MALDI MSI technique is widely recognized for its significant utility in the identification and visualization of metabolites and endogenous biomarkers. MALDI MSI and liquid chromatography-tandem mass spectrometry (LC-MS/MS) techniques were used to successfully identify and measure the spatial arrangement of tetrandrine within different organs of rats (Tang et al., 2019). The significance of MALDI MSI was underscored in its ability to facilitate drug metabolite visualization, quantification, and identification while preserving tissue integrity. In a comprehensive review conducted by Spruill et al. (2022) MALDI MSI was used for visualizing and quantifying drug metabolites and naturally occurring molecules within tissue samples of rat's whole-body, brain, lung, liver, kidney, stomach, and intestine. Moreover, Kaya et al. (2023) provided evidence supporting the efficacy of MALDI MSI as a valuable technique for the identification and spatial mapping of carboxyl and aldehyde metabolites found in brain tissues. These reports provided additional evidences to support the role of MALDI MSI in analyzing the spatial distribution of drugs within specific organs during ADME studies.

The renal organ assumes a crucial function in the processes of drug metabolism and elimination, rendering its susceptibility to the adverse effects of drug-induced toxicity (Bajaj et al., 2018). The LC-MS/MS study conducted by Zhao et al. (2022) demonstrated that febuxostat, a drug used to treat hyperuricemia, exhibited renal accumulation in rats with significant renal impairment following repeated administrations. Their findings revealed a plausible detrimental impact on individuals with pre-existing renal impairment. The MALDI MSI investigation of Jung et al. (2016) revealed notable alterations in metabolites within renal tissues subsequent to furosemide administration. The drug accumulation in renal tissues suggests a potential occurrence of drug-induced nephrotoxicity, thereby emphasizing the importance of drug optimization and redevelopment (Chan et al., 2020).

Rotenone, a bioactive compound derived from plants, has demonstrated significant anticancer properties by inducing apoptosis in MCF-7 cells, a human breast cancer cell line (Deng et al., 2010). The research conducted by Hu et al. (2016) unveiled the anticancer properties of rotenone as a potent inhibitor of complex I in the mitochondrial electron transport chain. This inhibition leads to the activation of NOX2 and subsequent generation of reactive oxygen species (ROS). Rotenone induced apoptosis in various cancer cell lines by selectively targeting cancer cells while preserving the integrity of the overall cellular machinery (Hu et al., 2016). Despite the significant anticancer properties, rotenone has an inherent toxicity and extensively used as an agricultural pesticide (Zhang et al., 2022). Therefore, a recent investigation conducted by Hernandez et al. (2023) illustrates the potential of rotenone as an anticancer drug by synthesizing its derivatives, which exhibited encouraging inhibitory effects on cell proliferation.

In this study, MALDI MSI was employed to investigate the spatial distribution and localization of rotenone, along with endogenous metabolites, in rat kidney tissues subsequent to a single oral administration. A methodical and systematic approach utilizing MALDI MSI was implemented to evaluate the spatial distribution patterns of rotenone within renal tissues of a drug-treated rat sample. The optimal workflow for MALDI MSI encompasses several key steps. These include the careful sectioning of kidney tissues, the application of matrix onto the tissue sections, and the subsequent acquisition and interpretation of MALDI MSI data. The spatial distribution and specific localization of rotenone and endogenous metabolites within the renal tissues of rats provided significant findings that can augment our understanding of the drug’s pharmacokinetics and early toxicity characteristics. These insights may also offer guidance for future investigations pertaining to the synthesis of rotenone derivatives, which hold promise as potential anticancer therapeutics.

## Materials and methods

### Drug

Rotenone (Sigma Aldrich, St. Louis, MO, United States) was mixed with pure corn oil (Marca Leon, Cagayan de Oro, Philippines) to a final concentration of 5 mg/mL and sonicated at 60°C to enhance the dissolution rate of rotenone. The light-sensitive rotenone solution was then covered with aluminum foil for subsequent animal testing.

### Animal study

The rotenone solution was administered orally to rats at 2 mg/kg, while control rats received no drug nor solvent at all. Rats were sacrificed 24 h after drug administration, and the kidney and liver organs were immediately removed, flash-frozen with liquid nitrogen, and stored at −80°C in a freezer for long-term storage. The administration and harvesting of organs were conducted by the Pharmalytics Corporation, accredited by the Food and Drug Administration (FDA, Philippines) for bioavailability and bioequivalence research. The animal study was reviewed and approved by the Institutional Animal Care and Use Committee (IACUC) Esteleydes Animal Laboratory and Research Facility and by the Bureau of Animal Industry—Animal Health and Welfare Division in the Philippines.

### Tissue sample preparation

Frozen kidney and liver samples were immediately embedded in a 3% agarose solution (3.0 g agarose in 97 mL distilled H_2_O) until fully covered. The cryo-molds were immediately placed in a −80°C freezer overnight to completely solidify the embedding medium (Costa et al., 2013; Marques et al., 2014; Dalisay et al., 2015; Suarez et al., 2023).

Agarose-embedded kidney and liver organs were cut longitudinally at 5 µm thickness at −20°C using a cryostat (Leica CM 1950; Leica Biosystems, Germany), and thaw-mounted onto clean glass slides. The agarose-embedded kidney or liver was trimmed up to 500 µm to remove the agarose covering a portion of the organ. Subsequently, seven layers from the outer portion towards the middle were identified with a 100 µm distance between each layer. Representative cryosections were obtained from each identified layer of the untreated and drug-treated rat kidney. The agarose surrounding the kidney or liver cryosectioned tissue was manually removed before mounting onto a glass slide. Prior to matrix coating, the glass slides mounted with thin kidney cryosections were placed in a box and kept in a vacuum desiccator cabinet overnight (Costa et al., 2013; Marques et al., 2014; Dalisay et al., 2015; Suarez et al., 2023).

Matrix solution was applied to evenly coat the entire tissue surface using a HTX M5 Sprayer (HTX Technologies, LLC, Carrboro, NC, United States). The parameter settings were optimized to avoid metabolite delocalization and compromised spatial integrity. The kidney sections were sprayed with a matrix solution of 2,5-dihydroxybenzoic acid (DHB) (Acros Organics, New Jersey, United States) at a concentration of 40 mg/mL in MeOH/H_2_O (1:1 v/v). Matrix solution was homogeneously applied for 6 passes in a criss-cross (CC) pattern with a nozzle temperature of 80°C, 1 mm track spacing, 1,250 mm/min nozzle speed, and 10 psi nitrogen pressure.

### MALDI MS and MS/MS imaging analysis, and ion mobility

The MALDI mass spectrometry imaging (MSI) of kidney tissue sections was complemented with ion mobility separation (MALDI HDMS Imaging) using the MALDI SYNAPT XS (Waters Corporation, Manchester, United Kingdom). The MALDI data was acquired using 280 laser energy, 1 s scan time per pixel, positive ion “resolution” analyzer, 1,000 Hz laser repetition rate, mass range of *m/z* 50–750, and 200 µm pixel resolution. Acquisition patterns were defined using the HDImaging software (v1.5, Waters Corporation, Manchester, United Kingdom) and the pattern file was imported to MassLynx (v4.2 Waters Corporation, Manchester, United Kingdom) for data acquisition. Red phosphorus (Sigma Aldrich, St. Louis, MO, United States) was used as the external calibrant over the same mass range of *m/z* 50–750 prior each run. Lock mass correction was performed every 600 s for 10 s by laser ablation of red phosphorus spotted off-tissue.

To further enhance the separation between closely structurally related molecules, all data acquired by MALDI MSI was complemented with ion mobility spectrometry (IMS). The IMS settings were as follows: drift time with a 650 m/s wave velocity and a wave height of 40 V on its T-Wave. The drift time was acquired at a starting wave velocity of 1,385 m/s and ending at 440 m/s to eliminate noise signal interferences.

The MALDI ion mobility MS/MS imaging was performed to identify the fragment ions of rotenone using the MALDI quadrupole time-of-flight (qTOF) SYNAPT XS High Definition Mass Spectrometer (HDMS) (Waters Corporation, Manchester, United Kingdom). The MALDI MS/MS and ion mobility was performed in the last part of the T-wave called “transfer cell.” The precursor ion (protonated adduct of rotenone, *m/z* 395.1) was subjected to collision-induced dissociation (CID) with a trap collision ramp set from 15 to 55 V and a transfer collision voltage of 28 V. Mass spectra were collected from *m/z* 50–750, 200 μm pixel size, and 1 s scan time. The ion mobility separation was displayed in DriftScope™ (v2.9 Waters) as a two-dimensional (2D) map (*m/z*:drift time) of rotenone and its fragment ions.

### Drug metabolite biotransformation

The structural profile of biotransformed rotenone metabolites was predicted using GloryX (Stork et al., 2020; de Bruyn Kops et al., 2021) and Biotransformer 3.0 (Djoumbou-Feunang et al., 2019; Wishart et al., 2022). A Simplified Molecular Input Line Entry System (SMILES) or an .sdf file of rotenone was initially created using ChemDraw Professionals program and uploaded to the free online tools for metabolite prediction. For GloryX metabolite prediction, rotenone was evaluated for Phase 1 and Phase 2 metabolism separately, while for Biotransformer 3.0 metabolite transformation, rotenone was evaluated for Phase 1, Phase 2, and EC-based metabolism separately. The cytochrome (CYP)-mediated metabolism, clearance, and half-life of rotenone were predicted using ADMETlab 2.0 (https://admetmesh.scbdd.com) (Xiong et al., 2021).

### Renal metabolite biomarkers

To identify possible metabolic alterations, the *m/z* values of endogenous compounds found in kidney were extracted from the human metabolome database (http://www.hmdb.ca). The spatial mapping of endogenous compounds was visualized by MALDI HDMS imaging. The detection of metabolite biomarkers was supported by their spatial distribution, normalized ion intensity, *m/z* accuracy, and similar drift time bins in thin tissues sectioned from the seven identified layers of drug-treated and control rat kidney.

### Quantitative MSI (qMSI) analysis

The MALDI quantitative Mass Spectrometry Imaging (qMSI) analysis of rotenone was performed using an on-tissue spotting method (Schulz et al., 2019). This method utilized an untreated kidney tissue sample which was sectioned and mounted on a clean glass slide. An on-tissue calibration curve method for qMSI was followed (Kibbe and Muddiman, 2024). To investigate the linear range of the mass spectrometer, a calibration curve was generated by preparing a blank sample and nine calibrants at 5, 10, 20, 30, 40, 50, 70, 90, and 100 ng/μL via serial dilution of the rotenone standard solution in MeOH. Linear regression analysis was applied and inspected to confirm linearity. The untreated tissue was spotted with calibrants, allowed to dry, and coated with matrix (DHB 40 mg/mL in MeOH:H_2_O, 1:1 v/v). MALDI MSI data were acquired in 100 μm pixel resolution using MALDI SYNAPT XS (Waters Corporation, Manchester, United Kingdom) in positive mode. The experiment was performed with three replicates per calibrant in three independent trials. In order to address variations that may occur during data acquisition for qMSI, the ion signals were normalized to the total ion current (TIC). The intensities obtained for each concentration were plotted and approximated using the least-squares method. The limits of detection (LOD) and quantification (LOQ) were calculated by averaging the calibration curves, using signal-to-noise ratios (S/N) of 3:1 for LOD and 10:1 for LOQ. The validity of these limits was confirmed by applying serially diluted rotenone onto the tissue. In cases where the blank response has a standard deviation (SD) of zero, the SD of the lowest non-zero concentration was utilized. The SD value was divided by the slope of the calibration curve and multiplied by 3.3. The LOD was calculated using the formula

$$LOD=3.3\;x\;\left(\frac{{SD}_{lowest\;non-zero\;concentration}}{slope\;of\;the\;calibration\;curve}\right)$$

In a similar manner, the LOQ was evaluated by performing a series of calculations. The LOQ was determined according to the formula

$$LOQ=10\;x\;\left(\frac{{SD}_{lowest\;non-zero\;concentration}}{slope\;of\;the\;calibration\;curve}\right)$$

To calculate the LOD and LOQ values in kidney tissue (pg/ng), the values were divided by the renal tissue density (ng/µL). The tissue density was calculated by measuring the weight of the glass slide with the tissue, subtracting the weight of a blank glass slide to find the tissue mass in milligrams, converting it to nanograms, and then determining the tissue volume using the equation: volume = length × width × thickness × 0.523 (Melchioretto et al., 2016).

### Tissue staining

Matrix was removed from the laser-ablated kidney sections using 95% EtOH for 20 s before rehydration with distilled water. The tissue-mounted glass slides were immersed in Harris hematoxylin solution (Sigma Aldrich, St. Louis, MO, United States) for 4 min and the excess was rinsed off with deionized water twice for 1 min. Hematoxylin differentiation of tissues was then performed using 0.5% acid alcohol for 10 s. The glass slides were rinsed using deionized water for 1 min followed by soaking in ammonia solution for 1 min and rinsing in deionized water twice for 1 min. Kidney sections were then counterstained using eosin Y solution (Sigma Aldrich, St. Louis, MO, United States) for 2 min. Lastly, samples were immersed in 95% ethanol solution twice for 2 min and 100% ethanol solution once for 2 min (Ramírez et al., 2020). All glass slides with tissue samples were air dried prior to histology analysis.

### Data processing

MALDI HDMS raw data was imported into High-Definition Imaging (HDI) software v1.5 (Waters Corporation, Manchester, United Kingdom). Data processing was performed by detecting 10,000 intense peaks between a mass range of *m/z* 50–750, a drift time maximum of 200 bins, and a lock mass-based correction at *m/z* 464.6064. Following data processing, peak picking was carried out and data was normalized based on the total ion current (TIC). The spatial distribution of rotenone and kidney endogenous molecules from the *m/z* peak list was visualized using the HDI software. The ppm error cutoff value for detected *m/z* peaks <6 ppm, while the drift time difference must be ±1 bin between the experimental groups and that of the rotenone standard drug spotted off-tissue. The ion intensity of ion images was displayed using the Weather1 gradient at a linear scale composition for MALDI MSI and inverted Weather1 gradient at log scale composition for MALDI tandem MS Imaging. The tentative identification and assignment of kidney metabolites were based on the metabolites in the human metabolome database (http://www.hmdb.ca). The *m/z* values and structure of these renal endogenous molecules were verified using PubChem (https://pubchem.ncbi.nlm.nih.gov), ChemDraw Professionals, and by the related literature (Jung et al., 2016; Nizioł et al., 2020; Zhu Y. et al., 2022).

### Statistical analysis

A multivariate analysis was performed by importing the merged CSV file of processed MALDI image data into EZinfo v3.0 (Waters Corporation, Manchester, United Kingdom). Principal component analysis (PCA) was used to find patterns or trend in the MALDI dataset of the control and drug-treated groups. A peak list of biomolecules was manually created for PCA analysis from the laser-ablated kidney tissue sections. Pareto variance was used to scale the x-variable and to eliminate any bias in this unsupervised algorithm. Similar and far away observations between the dataset were illustrated based on the t[1] and t[2] indices. Meanwhile, the statistical differences between the mass ion intensity of endogenous compounds in two treatment groups were analyzed using the Student’s t-test of GraphPad Prism version 9.4.0 (GraphPad Software Inc., San Diego, CA, United States). The significance threshold was set at *p* < 0.05 (*), *p* < 0.01 (**), and *p* < 0.001 (***).

## Results and discussion

### MALDI MS, ion mobility, and MS/MS imaging analysis of rotenone

**FIGURE 1:**
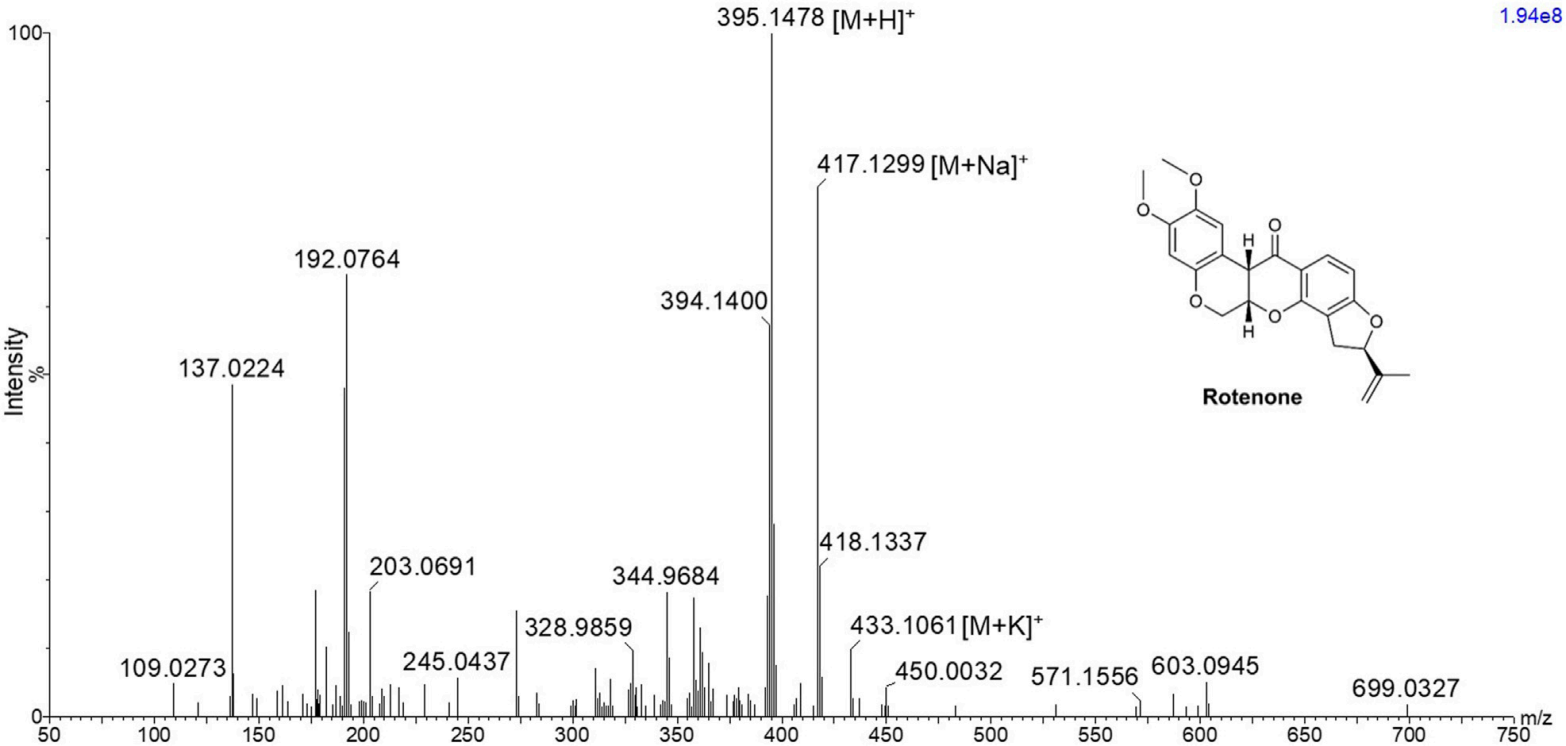
MALDI MS spectrum of rotenone showing *m/z* 395.1478 [M+H]^+^, 417.1299 [M+Na]^+^, and 433.1061 [M+K]^+^ using 2,5-dihydroxybenzoic acid matrix solution with a concentration of 40 mg/mL in MeOH:H_2_O (1:1 v/v).

In order to investigate the ionization potential of rotenone using MALDI MS, a solution of rotenone reference drug [2 μg/μL in MeOH:H_2_O (1:1 v/v)] and DHB [40 mg/mL in MeOH:H_2_O (1:1 v/v)] matrix was spotted onto a stainless MALDI plate. The MALDI mass spectra of rotenone (exact mass 394.1416 Da) presented a protonated ion [M+H]^+^ as the major adduct ion at *m/z* 395.1478 (Figure 1). Furthermore, the parent drug’s sodium and potassium adduct ions were also found at *m/z* 417.1299 [M+Na]^+^ and 433.1061 [M+K]^+^, respectively.

**FIGURE 2:**
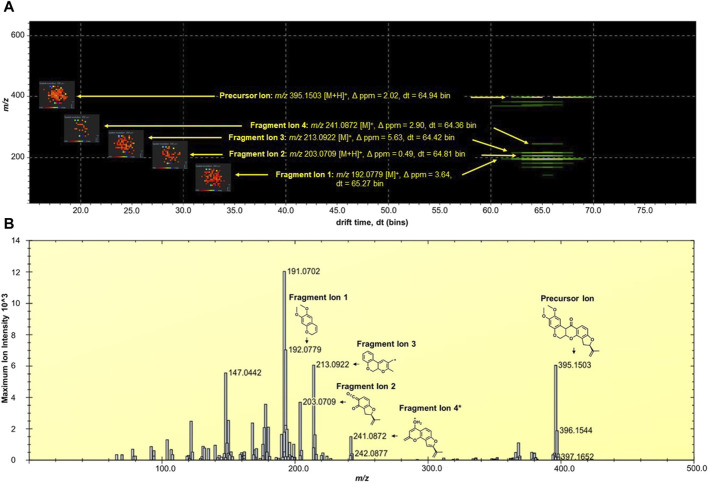
**(A)** DriftScope display of the two-dimensional (2D) map of (*m/z*:drift time) of rotenone and its fragment ions. The 2D map contained the parent drug precursor and fragment ions, which were aligned vertically at 64.7 ± 0.3 drift time (dt) bins. The MALDI ion images were placed on the left side of the precursor and fragment ions. The MALDI MS images were acquired at 200 µm spatial resolution and processed using the inverted Weather1 gradient and log scale image composition. **(B)** MALDI MS/MS Imaging spectrum of rotenone generated by the HDImaging software (v1.5 Waters Corporation, Manchester, United Kingdom). The precursor and fragment ions were annotated and the mass error was calculated. *Proposed structure is based on the predicted fragment of CFMID (https://cfmid.wishartlab.com/) which matches the elemental composition, DBE, and ppm error provided by the MassLynx software (v4.2 Waters Corporation, Manchester, United Kingdom).

The MALDI HDMS/MS imaging analysis showed the fragment ions of rotenone in the two-dimensional (2D) map of drift time and *m/z* peaks. Ion mobility separation was carried out in DriftScope to validate if the fragment ions belong to the parent drug. The fragment ions have vertical drift time alignment with the drug precursor (Figure 2A). The drift time of fragment ions was observed between 64.36 and 65.27 bins. These fragment ions share similar drift times, suggesting the correct assignment of fragment ions to their parent precursor. It is interesting to point out that there were isobaric *m/z* peaks observed in the MALDI HDMS/MS imaging that could be linked to the fragmentation of rotenone. For instance, the MALDI HDMS/MS imaging analysis revealed an isobaric peak (*m/z* 192.0750) similar with the main fragment ion (*m/z* 192.0779) of rotenone (Supplementary Figure S1). However, it is worth mentioning that the drift time of this isobaric peak did not match with the precursor ion rotenone (Supplementary Figure S1). In the MALDI ion mobility MS/MS technique, the sorting of isobaric ions takes place initially, followed by their fragmentation in the transfer cell. The analysis of their *m/z* values is then carried out in the orthogonal-TOF. Therefore, the separation of drift time remains intact following dissociation, ensuring that all fragment ions originating from the same precursor ion exhibit similar drift times. Figure 2B displays the plausible fragment ions of rotenone observed in the MALDI mass spectrum with *m/z* values of 192.0779, 203.0709, 213.0922, and 241.0872, and their corresponding proposed structures. Notably, the fragment ions obtained using MALDI HDMS/MS imaging displayed comparable MS images in terms of spatial resolution and spatial distribution (Figure 2A). This observation strongly suggests that these fragment ions are indeed associated with rotenone. In addition, our MALDI HDMS/MS data aligns with the HPLC MS/MS data from previous studies (Cordaro et al., 2004; Caboni et al., 2008; Rhee et al., 2016). These findings represented the first report of rotenone fragment ions detected by combining ion mobility separation with MALDI MS/MS imaging.

### Metabolite analysis and drug detection in kidney tissues

The kidney functions as the primary organ within the excretory system and assumes a significant role in eliminating xenobiotics. The structure comprises multiple tissue regions, namely the cortex, medulla, pelvis, and hilum (Radi, 2019). Following the oral rotenone administration at a dosage of 2 mg/kg for a duration of 24 h, a qualitative MALDI MSI analysis was conducted to evaluate the spatial distribution of rotenone and its metabolites within tissue sections of rat kidney.

Rotenone, an isoflavone compound, is abundant in the roots of various plant species and possesses a high degree of lipophilicity. The compound’s inherent lipid solubility facilitates its unrestricted passage across biological membranes, irrespective of transporter molecules, leading to its accumulation within cellular organelles, particularly mitochondria. This accumulation has the potential to interfere with cellular pathways, as suggested by previous studies conducted by Wu et al. (2020) and Uversky (2004). According to Ray (1991), the LD50 (median lethal dose) of rotenone when administered orally to rats was found to vary between 25 and 132 mg/kg body weight.

In a study conducted by Jiang et al. (2017), the group examined the potential toxicological impacts of rotenone by assessing various aspects including morphological changes, biochemical alterations, oxidative stress-related responses, and modifications in apoptotic factors within the renal tissue of rats. They documented that the oral acute toxicity of rotenone in rats was observed at a dosage of 34.10 mg/kg body weight. Additionally, they conducted morphological and biochemical analyses, which revealed kidney tissue damage. Furthermore, the research conducted by Jiang et al. (2017) demonstrated that rotenone administration resulted in oxidative harm to kidney tissues. This was evidenced by the elevated concentrations of glutathione, malonaldehyde, and ROS. These findings suggest a correlation between the induction of renal tissue apoptosis through the mitochondrial pathway and the observed oxidative damage caused by rotenone. Interestingly, no reports so far have documented the effect of rotenone in kidney endogenous molecules and its potential implications for the prognosis of kidney injury or disease. This indicates a gap in the current understanding on the mechanism of how rotenone contributes to renal injury and highlights the need for further research to investigate potential biomarkers for renal injury.

In anticipation that there will be several isobaric *m/z* peaks arising from the matrix and endogenous metabolites, ion mobility spectrometry was performed to allow another level of ion separation and compound-specific detection in kidney tissues. Hence, the ion mobility of rotenone as reference drug was profiled. It was observed that rotenone has a drift time between 66 and 67 bins. This value (±1 bin) was used as a guide apart from the *m/z* error obtained in mass spectrometry for the succeeding investigations of kidney tissues.

**FIGURE 3:**
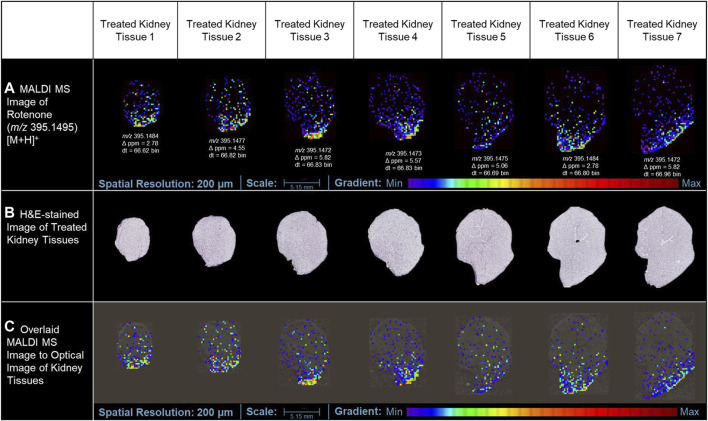
MALDI MS imaging analysis of rotenone distribution in drug-treated kidney tissue sections. **(A)** MALDI MS images of the protonated adduct [M+H]^+^ of rotenone in representative tissues from different layers of treated kidney tissues, **(B)** H&E of rotenone-treated kidney tissue sections, **(C)** MALDI MS images of the protonated adduct [M+H]^+^ of rotenone overlaid on the optical image of kidney tissues. The MALDI MS images were normalized based on their TIC.

In this study, the utilization of MALDI MSI allowed the visualization of the specific distribution of the drug and the quantification of the ion intensity of the protonated parent drug (*m/z* 395.1495 [M+H]^+^) with a high degree of accuracy in the renal cortical region (as depicted in Figure 3A). Although the ion intensities observed were relatively low, the spatial distribution of the protonated rotenone exhibited a comparable pattern across representative tissues from seven distinct layers of the kidney. MALDI HDMS/MS imaging was performed to validate the presence of the parent drug (*m/z* 395.1495 [M+H]^+^) in the treated kidney tissue. Unfortunately, the ion intensity was insufficient for a successful MS/MS analysis. The signal from the parent drug proved to be too weak that is challenging to detect and analyze further, resulting in suboptimal fragmentation and unreliable MS/MS spectra. To address this issue, we validated our MALDI MS imaging data using ion mobility spectrometry. The detected ion at *m/z* 395.1495 [M+H]^+^ was subsequently confirmed as rotenone, based on its consistent drift time (66.7 ± 0.3 bins) compared to the rotenone reference drug. This additional validation step provided confidence in the identification of the parent drug on treated kidney tissues despite the challenges encountered in the MS/MS analysis. Intriguingly, an ion with *m/z* 395.1495 [M+H]^+^ was also observed in the control tissue, potentially indicating the presence of rotenone. Nevertheless, after conducting a thorough analysis, it was observed that the drift time of this ion did not match with that of the rotenone standard. These findings highlight the importance of ion mobility spectrometry in compound characterization, which complements the analysis conducted through MALDI MSI (Supplementary Figure S2). The drug-treated kidney tissues were stained with hematoxylin and eosin (H&E) (Figure 3B). The optical image of kidney cryosections was overlaid by the MALDI MS images of the protonated rotenone (Figure 3C). The sodiated adduct of rotenone was also detected and has a similar spatial distribution with the protonated adduct in the renal cortex. The primary origin of sodium, a crucial electrolyte, is the reabsorption mechanism predominantly taking place in the proximal tubules of the cortex (Radi, 2019; Shrimanker and Bhattarai, 2023).

In addition to the minimal drug detection in MALDI MSI, the use of a conductive slide may also have a negative impact on the low-level ion intensity of rotenone detected in tissue sections treated with the drug. In tissue cryosectioning, several literatures reported that non-conductive substrates could provide similar MALDI imaging results with that of the conductive platforms. In the study of Garrett et al. (2007), non-conductive and conductive substrates provided comparable MS images of phospholipids using an intermediate-pressure MALDI linear ion trap mass spectrometer. In the review paper of Dong et al. (2016), orthogonal MALDI-TOF systems did not require conductive surfaces for successful analyte ionization, and the utilization of regular glass slides is acceptable. Due to the orthogonal geometry of the instrument, the ion source is decoupled with the mass analyzer, eliminating the need to accelerate ions directly from the source to the mass analyzers (Prentice and Spraggins, 2021). This lowers the electric potential needed to extract ions in the sample surface, allowing for effective extraction of ions in non-conductive substrates.

Our attempts to validate the presence of rotenone in tissues using LC-MS for conclusive evidence were not successful. The sample did not show any presence of rotenone ions. It is possible that the variation in the findings could be attributed to the potential deterioration of the specimen, which had been embedded in agarose and preserved for over 3 years before the LC-MS investigation. It is plausible that the sample underwent chemical changes and compound degradation, which could have impacted the detection and quantification by LC-MS. Furthermore, several factors, including the low concentration of rotenone, the presence of other compounds influencing the matrix, and potential challenges with ionization efficiency, may have contributed to the absence of detection. The challenges highlighted in this study emphasize the sample integrity, and the subsequent influence they have on the accuracy of LC-MS analysis. Nonetheless, our analysis using MALDI MSI produced conclusive findings. We successfully identified and mapped the presence of rotenone in the treated tissues, which was confirmed by high-resolution mass spectrometry and ion mobility separation (Figure 3).

The kidney cortex is highly susceptible to xenobiotics due to its significant exposure to approximately 80% of the total blood flow directed towards the renal system (Anders, 1980; Radi, 2019). Studies on the binding of [3H]-dihydro-rotenone to different tissues have demonstrated its highest accumulation in the kidney and heart (Organization for Economic Cooperation and Development, 2020). Although the spatial distribution was not extensively discussed in these studies, our MALDI MSI findings revealed that rotenone accumulates at the lower boundary of the kidney cortex. This suggests that the distribution of rotenone is not uniform and is instead concentrated in specific areas. Our study also highlighted how MALDI MSI presents high-resolution ion images depicting the detection of protonated adduct of rotenone within kidney tissue sections. While we primarily rely on MALDI MSI for this observation, this localized accumulation of rotenone in the renal cortex can be attributed to the kidney’s metabolic physiology. The cortex relies on a significant blood flow to carry out its crucial filtration process. However, this also means that the cortex is constantly exposed to various substances (xenobiotics) found in the bloodstream (Faria et al., 2019), such as rotenone. Variations in blood flow within the renal cortex can lead to localized areas of higher drug concentration. Factors such as regional differences in vascular architecture, local oxygenation levels, and the presence of specific transporters can influence these variations (Vagabov et al., 2020; Edwards and Kurtcuoglu, 2022).

Furthermore, renal blood flow is not static but exhibits dynamic oscillations, which can further impact drug distribution. These fluctuations have the potential to influence the distribution of drugs within the body. For instance, the fluctuations in renal blood flow can cause changes in drug delivery and clearance in the cortex. An interesting example of this phenomenon is the vasodilator angiotensin II. Its distribution is not uniform and changes over time due to fluctuations in renal blood flow, as observed through laser speckle imaging (LSI) (Postnov et al., 2015). These oscillations may result in elevated drug concentrations in certain regions, which can heighten the likelihood of nephrotoxicity. Our findings underscore the complexity of drug distribution within the renal cortex and emphasizes the need for considering regional blood flow variations when evaluating nephrotoxic risks and drug efficacy.

In a previous investigation conducted by Nilsson et al. (2015), the application of MALDI MSI was elucidated to examine the comparative prevalence and spatial arrangement of polymyxin B1 and colistin within sections of rat kidney tissue. Polymyxins serves as a final resort for antibiotic treatment due to their nephrotoxicity, thus imposing limitations on drug administration. Rat samples were dosed with the drug subcutaneously and MALDI MSI data were collected from 14-µm thick kidney tissues at three different time points. The MALDI MSI analysis demonstrated that polymyxin B1 and colistin exhibited a predominant localization in the renal cortex region (Nilsson et al., 2015). Furthermore, their findings indicated that the accumulation of these drugs in the cortical region of the kidney increased proportionally with the duration of dosing.

### Biotransformed metabolites of rotenone

Multiple scientific investigations have documented comprehensive characteristics pertaining to drug metabolism within the renal system. The renal system employs metabolizing enzymes, specifically cytochrome P450 (CYP) and non-P450 enzymes, to facilitate the metabolism and elimination of both endogenous and exogenous substances. This process occurs through two distinct phases of metabolism, namely Phase 1 and Phase 2. These phases involve a series of reactions, including oxidation, reduction, hydrolysis, and conjugation, which contribute to the overall metabolism and clearance of compounds (Anders, 1980; Bajaj et al., 2018).

The Phase I metabolic profile of rotenone resulted to the identification of five metabolizing CYP enzymes: namely, CYP1A2, CYP3A4, CYP2D6, CYP2C9, and CYP2C19 (Table 1). Based on the evaluation metabolic ranges, ADMETlab 2.0 suggested that rotenone is a substrate and a non-inhibitor of CYP1A2. Meanwhile, rotenone could act as a substrate and inhibitor of CYP3A4, CYP2D6, CYP2C9, and CYP2C19. These cytochrome P450 enzymes could facilitate Phase I biotransformation of rotenone into water-soluble derivatives. Aside from evaluating metabolic properties, ADMETlab 2.0 provided results on the possible clearance and half-life of a drug. Rotenone has a calculated clearance value of 8.641 mL/min/kg, which may indicate moderate clearance mechanisms (Xiong et al., 2021). Rotenone has a predicted half-life of less than 3 h, suggesting a possible need of frequent dosage to achieve its desired effect and exposure (Smith et al., 2018).

**TABLE 1:** CYP-metabolizing enzymes predicted by ADMETlab 2.0.

CYP prediction		Metabolic evaluation range	Interpretation
CYP1A2	Substrate	0.9–1.0	Rotenone could be a substrate and non-inhibitor of CYP1A2. (The output value is the probability of being substrate/inhibitor, within the range of 0–1.)
	Inhibitor	0.1–0.3	
CYP3A4	Substrate	0.5–0.7	Rotenone could be a substrate and inhibitor of CYP3A4. (The output value is the probability of being substrate/inhibitor, within the range of 0–1.)
	Inhibitor	0.9–1.0	
CYP2D6	Substrate	0.9–1.0	Rotenone could be a substrate and inhibitor of CYP2D6. (The output value is the probability of being substrate/inhibitor, within the range of 0–1.)
	Inhibitor	0.5–0.7	
CYP2C9	Substrate	0.9–1.0	Rotenone could be a substrate and inhibitor of CYP2C9. (The output value is the probability of being substrate/inhibitor, within the range of 0–1.)
	Inhibitor	0.7–0.9	
CYP2C19	Substrate	0.7–0.9	Rotenone could be a substrate and inhibitor of CYP2C19. (The output value is the probability of being substrate/inhibitor, within the range of 0–1.)
	Inhibitor	0.9–1.0	

The major biotransformed products of rotenone were plausibly metabolized as hydroxylated conjugates. Among the CYP450 enzymes, the CYP3A4 and CYP2C9 were identified as the primary metabolizing enzymes in forming 12-αβ-hydroxy products *in vivo* (Caboni et al., 2004). Based on their findings, CYP3A4 demonstrated the highest activity in inducing rotenone metabolism, while CYP2C9 was determined as a highly site-specific metabolizing enzyme.

**FIGURE 4:**
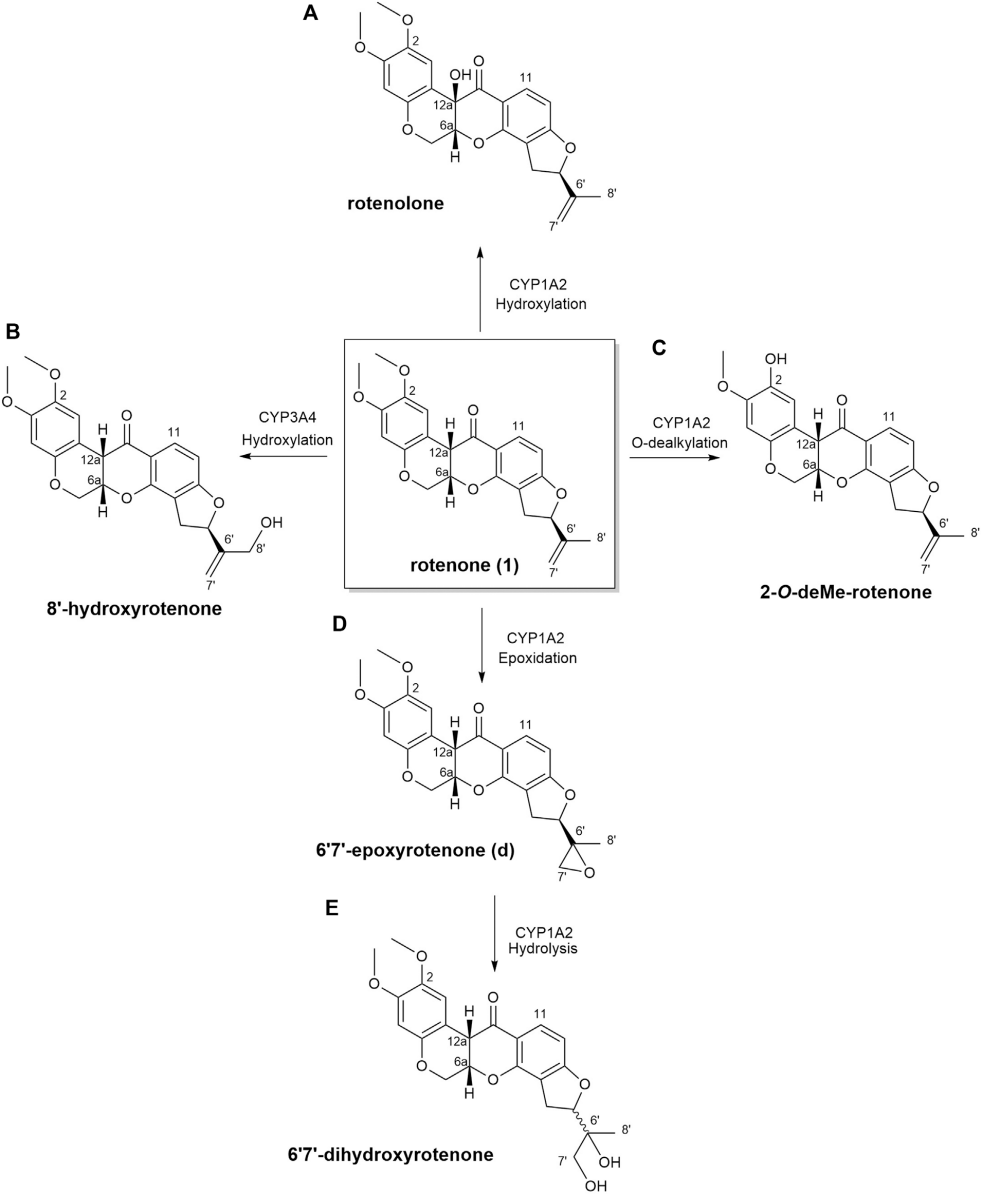
Biotransformation of rotenone predicted using GloryX and Biotransformer 3.0 computational tools. The chemical reaction and the plausible CYP450-metabolizing enzymes involved in the biotransformation of rotenone were further predicted by Biotransformer 3.0. Rotenone yielded **(A)** rotenolone and **(B)** 8’-hydroxyrotenone through the hydroxylation reactions metabolized by CYP1A2 and CYP3A4 enzymes, respectively. The O-dealkylation of rotenone with CYP1A2 resulted to the formation of **(C)** 2-O-deMe-rotenone. CYP1A2 initiated the formation of **(D)** 6’,7’-epoxyrotenone through an epoxidation reaction, and the hydrolysis of its 6’,7’ position led to the formation of **(E)** 6’,7’-dihydroxyrotenone.

In this current investigation, the anticipated biotransformation of rotenone due to cytochrome P450-mediated metabolism was predicted using GloryX and BioTransformer 3.0 software (as depicted in Figure 4). The specific metabolic biotransformation of rotenone in human subjects has yet to be thoroughly investigated. In our study, GloryX and Biotransformer 3.0 have generated predictions of rotenone metabolites that were previously observed in various animal species, including rats, fish, and mice (Fukami et al., 1967; Fukami et al., 1969). The biotransformation products of rotenone have been reported to involve specific chemical reactions. One such reaction is the hydroxylation of an alicyclic tertiary carbon at the 12*a*-position, resulting in the formation of a compound called rotenolone (C_23_H_22_O_7_, with an exact mass of 410.1366 Da and a molecular ion peak [M+H]^+^ observed at *m/z* 411.1444). Another reaction involves the oxidation of the isopropenyl side chain at the 8′position, leading to the formation of a compound known as 8′-hydroxyrotenone (C_23_H_22_O_7_, with an exact mass of 410.1366 Da and a molecular ion peak [M+H]^+^ observed at *m/z* 411.1444). Additionally, the oxidation of the same side chain at the 6′,7′positions results in the formation of a compound called 6′,7′-dihydroxyrotenone (C_23_H_24_O_8_, with an exact mass of 428.1471 Da and a molecular ion peak [M+H]^+^ observed at *m/z* 429.1549), agreeing with previous studies (Fukami et al., 1969; Caboni et al., 2004). Furthermore, a metabolite that corresponds to the *O*-demethylation at the 2-position (chemical formula: C_22_H_20_O_6_, exact mass: 380.1260 Da, [M+H]^+^ ion at *m/z* 381.1338) has been documented in a previous study (Caboni et al., 2004). The previously mentioned metabolites, along with all anticipated metabolites, were employed as a reference for the identification and quantification of their spatial distribution within kidney tissues.

In this study, no biotransformed metabolites of rotenone were detected in drug-treated kidney tissue sections using MALDI MSI. Further MALDI MSI analysis revealed no biotransformed metabolites in liver tissues of a rotenone-treated rat sample. It is known that rotenone has a high metabolic clearance rate of approximately 100 mL/min/kg (Udeani et al., 2001; Organization for Economic Cooperation and Development, 2020), which may indicate rapid metabolism. Moreover, the water-soluble products of rotenone metabolism were most likely excreted quickly from the body due to the drug’s short half-life (Smith et al., 2018). Consequently, this pharmacokinetic property of rotenone might explain why we have not detected any traces of rotenone metabolites in the liver and kidney 24 h following drug intake. However, the results depicted in Figure 4 revealed the potential biotransformed products of rotenone metabolism, which were analyzed using advanced predictive modeling tools. A urine analysis using LC-MS is necessary to definitively determine the existence of water-soluble metabolites of rotenone. This additional step will not only serve to validate findings in our future research, but it will also offer invaluable insights into the metabolic fate of rotenone *in vivo.*

### Renal biomarkers of altered metabolism

Although MALDI images revealed clear spatial localization of rotenone metabolites in the kidney, no discernible histological alterations were detected in kidney tissue sections stained with hematoxylin and eosin (H&E). This discovery implies that the histological damage caused by a single dose of rotenone in the kidney may not be as significant as the toxic effects of polymyxin B1 and colistin when administered repeatedly (Nilsson et al., 2015).

Rotenone has an LD50 of 60 mg/kg when administered orally to rats (Jiang et al., 2017). Neurotoxic reactions were observed within a few minutes of exposure upon drug administration (Jiang et al., 2017). In another study, the repeated intragastric administration of rotenone at 2 mg/kg induced kidney tissue damage based on the increased levels of blood urea nitrogen (BUN), uric acid, and creatinine (Udeani et al., 2001). Vacuolar degeneration occurred in the tubular epithelial cells based on the histopathological analysis of rotenone-treated kidney tissue (Udeani et al., 2001). The said concentration of rotenone promoted vacuole formation in the nucleus and minimal inflammatory cell infiltration in the renal tissue. Moreover, rotenone induced oxidative stress by lowering the superoxide dismutase (SOD) activity in renal tissues (Jiang et al., 2017). The significant decrease in SUD activity (*p* < 0.01) could facilitate the rapid accumulation of free radicals.

**FIGURE 5:**
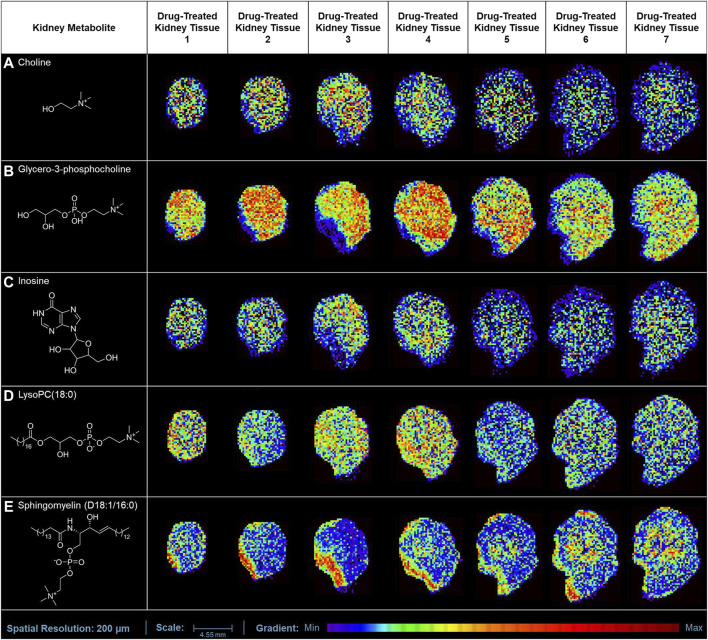
MALDI MS imaging analysis of kidney biomarkers and their corresponding spatial distribution in rotenone-treated kidney tissue sections. MALDI MS images of **(A)** choline (*m/z* 104.1071 [M+H]^+^), **(B)** glycero-3-phosphocholine (*m/z* 296.0665 [M+K]^+^), **(C)** inosine (*m/z* 307.0445 [M+K]^+^), **(D)** LysoPC(18:0) (*m/z* 562.3275 [M+K]^+^), and **(E)** sphingomyelin (D18:1/16:0) (*m/z* 703.5754 [M+H]^+^) across treated kidney tissue sections (5 µm thickness).

In our study, the one-time oral administration of rotenone (2 mg/kg) demonstrated an initial metabolic response on possible prognosis biomarkers for drug-induced kidney injury. Our MALDI MSI analysis revealed that ion intensities of endogenous compounds differed in the two experimental groups after 24 h. These kidney endogenous metabolites were involved in various metabolic processes indicative of possible early signs of renal injury. The utilization of emerging imaging technologies such as MALDI MSI enabled the detection and characterization of tissue biomarker candidates for rotenone-induced kidney injury (Figure 5). The MALDI MS images depicted the spatial arrangement of naturally occurring compounds, including organonitrogen compounds (Supplementary Figure S3), carboxylic acids and derivatives (Supplementary Figure S4), purine nucleosides (Supplementary Figure S5), and glycerophospholipids and sphingolipid (Supplementary Figure S6).

**FIGURE 6:**
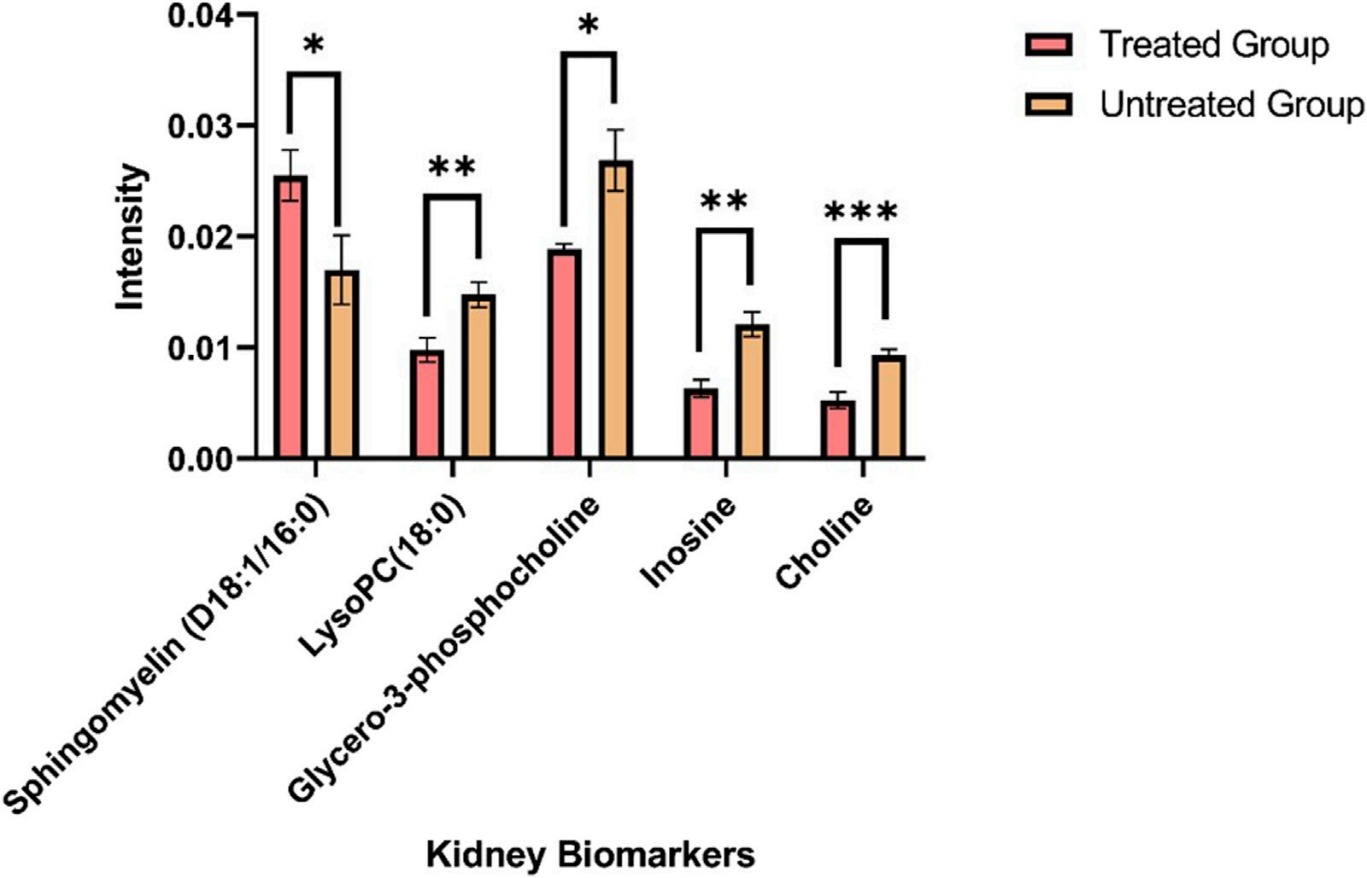
Mass ion intensity of kidney metabolites detected using MALDI MS Imaging. Each bar corresponded to the mean ion intensity of 7 tissue sections of untreated and rotenone-treated kidney where kidney biomarkers displayed statistical differences (*p < 0.05; **p < 0.01; ***p < 0.001) between the two experimental groups. The mean intensities were calculated from the ion intensities of kidney metabolites normalized by their total ion current (TIC).

The ion intensities of small-molecule renal metabolites of the drug-treated group were compared with that of the untreated group 24 h after a single oral administration. In rotenone-treated group, the ion intensity levels of sphingomyelin (D18:1/16:0), LysoPC(18:0), glycero-3-phosphocholine (GPC), inosine, and choline showed significant differences when compared with the control group (Figure 6; Table 2). Whereas several renal endogenous metabolites displayed differential ion intensities but without statistical differences between treated and control groups (Supplementary Table S1). The metabolic profile of these endogenous compounds may still represent as early signatures for possible kidney damage induced by drug treatments.

**TABLE 2:** Metabolite alteration in kidney tissues dosed with rotenone.

Kidney metabolite	HMDB ID	Formula	Adduct ion	Theoretical *m/z*	Measured *m/z*	Delta ppm	Ion intensity in rotenone-treated kidney tissues
Choline	HMDB0000097	C_5_H_13_NO	[M+H]^+^	104.1071	104.1071	0.96	↓***
Glycero-3-phosphocholine	HMDB0000086	C_8_H_20_NO_6_P	[M+K]^+^	296.0665	296.0659	2.03	↓*
Inosine	HMDB0000195	C_10_H_12_N_4_O_5_	[M+K]^+^	307.0445	307.0437	2.47	↓**
LysoPC(18:0)	HMDB0010384	C2_6_H_54_NO_7_P	[M+K]^+^	562.3275	562.3250	4.45	↓**
Sphingomyelin (D18:1/16:0)	HMDB0010169	C_39_H_79_N_2_O_6_P	[M+H]^+^	703.5754	703.5723	4.41	↑*

↑ indicates higher ion intensity level than that of the control group; ↓ indicates lower ion intensity level than that of the control group.

*p < 0.05; **p < 0.01; ***p < 0.001.

The overview of the variations between the two experimental groups was evaluated using principal component analysis (PCA). The PCA score plot demonstrated the distribution of kidney metabolites categorized into different classes using two principal components [t1] and [t2] (R^2^X[1] = 0.7227; R^2^X[2] = 0.2694) (Supplementary Figure S7). Phosphorylcholine (*m/z* 184.0733 [M]^+^), an organonitrogen compound, separated from the other metabolites inside the Hoteling’s ellipse. Furthermore, the PCA analysis of renal biomarkers with significant alterations in ion intensity levels revealed that notable differences between the two experimental groups (Supplementary Figure S8).

The presence of organic osmolytes, including choline and GPC, was observed in kidney tissues, exhibiting a distinct pattern of spatial distribution primarily concentrated in the renal medulla. Choline serves as a crucial precursor in the process of synthesizing phospholipids found in cellular membranes, such as GPC, LysoPC, and sphingomyelin (Tayebati and Amenta, 2013). In response to physiological stress, intracellular osmolytes undergo accumulation within the renal medulla (Garcia-Perez and Burg, 1991). In contrast, the ion intensity observed for choline has exhibited a significant decrease (*p* < 0.001) in kidney tissues treated with rotenone.

The cellular membrane is comprised primarily of signaling molecules known as glycerophospholipids. Our MALDI MSI analysis demonstrated that glycero-3-phosphocholine (GPC) and lysophosphatidylcholine (LysoPC) were the most abundant glycerophopholipids in kidney tissues. Glycero-3-phosphocholine was the predominant osmolyte in the medullary cells of rotenone-treated kidney. The choline concentration is primarily observed in tissues as GPC (Klein et al., 1993), which is the predominant metabolite found in the renal medulla of kidneys treated with rotenone. The GPC level was decreased significantly (*p* < 0.05) in drug-treated kidney tissues, which could be associated with osmotic imbalance in the renal medulla (Wei et al., 2014). The abnormal levels of GPC and choline were observed in the renal medulla of rats with lithium-induced insipidus (Bedford et al., 2008) and furosemide treatment (Jung et al., 2016). The altered osmotic regulation in the kidney may contribute to renal dysfunction and possible tissue damage.

Meanwhile, the presence of LysoPC in the kidney has been found to contribute to receptor-dependent proliferation, inflammation, and fibrosis, which can indicate renal injury (Chen et al., 2005; Ghazarian et al., 2013). LysoPC may be a potential target for inflammatory treatment due to its biological roles in ion channel activation, pro-inflammatory response, apoptotic induction, and oxidative stress (Liu P. et al., 2020). In our study, we found that palmitoyl-lysophosphatidylcholine [LysoPC(16:0)], stearoyllysophosphatidylcholine [LysoPC(18:0)], oleoyl-lysophosphatidylcholine [LysoPC(18:1)], linoleoyllysophosphatidylcholine [LysoPC(18:2)], and arachidonoyl-lysophosphatidylcholine [LysoPC(20:4)] were the major lysophosphatidylcholines that are widely distributed in kidney tissues. These lysophosphatidylcholines were also identified as the primary LysoPC in the human plasma (Liu P. et al., 2020). The ion intensity levels of LysoPC(18:0) decreased significantly (*p* < 0.01) in rotenone-treated kidney tissues. In a study conducted by Yoshioka et al. (2022), LysoPC(18:0) was identified as a potential target in designing new treatments for diabetic kidney disease. LysoPC(18:0) level was dysregulated in individuals with acute liver failure (Trovato et al., 2023). The significant decrease in the ion intensities of LysoPC(18:0) might be due to the inactivation of lysophosphatidylcholine acyltransferase (LPCAT) or phospholipase A2, which are crucial for converting phosphatidylcholine into LysoPC (Süllentrop et al., 2002). The ion intensity levels of LysoPC(P-16:0), LysoPC(16:0), LysoPC(16:1), LysoPC(17:0), LysoPC(18:1), LysoPC(18:2), and LysoPC(20:4) have minimal alterations in drug-treated kidney tissues, but without any statistical difference. These findings demonstrated that LysoPC species may serve as initial biomarkers for the onset of tissue damage in the kidney.

Sphingolipids are essential molecules where alterations in their metabolic pathways can be associated with renal injuries. In our study, sphingomyelin (D18:1/16:0) was locally distributed in the outer medullary and cortical regions of the kidney. The significant increase (*p* < 0.05) in sphingomyelin (D18:1/16:0) level was indicative of potential renal dysfunction. The elevated level of sphingomyelin (D18:1/16:0) could be positively correlated with renal impairment and various pathological conditions, including inflammatory disorders (Mallela et al., 2022), type-1 diabetes (Pongrac Barlovic et al., 2020), and malignancies (Jones et al., 2014).

Inosine, a nucleoside intermediate, serves as a pivotal component within the metabolic pathway of purines. The significant reduction of inosine level (*p* < 0.01), a vital regulator of inflammatory responses and oxidative stress, could potentially be linked to the early physiological responses to renal injury (Kim and Jo, 2022), as evidenced in rats displaying renal fibrosis (Liu H. et al., 2017).

Metabolite biomarkers that manifest at an early stage have been identified as significant factors in assessing the severity of renal dysfunction (Bergman et al., 2019; Nizioł et al., 2020; Lim et al., 2023a) within diseased kidney tissues. Untargeted metabolomics employs the technique of LC-MS for analysis (Lim et al., 2023b), whereas MALDI MSI provides distinct ion images that reveal the distribution and localization of metabolites within complex biological samples (Nizioł et al., 2020). MALDI MSI enables the spatial localization of metabolites, allowing for the visualization of the impact of drug absorption on the modulation of metabolic pathways within biological tissues. The enhancement of tissue metabolite detection is achieved through ion mobility separation. This complementary technique demonstrated a higher level of spatial resolution and drift time detection of target metabolites. The perturbed concentrations of renal endogenous metabolites have been linked to compromised renal metabolism and may serve as signatures of early stages of kidney injury (Jung et al., 2016). The metabolite profiles of small-molecule biomarkers using MALDI MSI has the potential to enhance drug localization and metabolism studies during the pre-clinical phases of drug development.

### MALDI quantitative MSI analysis

In recent years, the utilization of MALDI MSI has been investigated as a method for achieving absolute quantification. However, this approach has encountered challenges in its implementation, likely attributable to the presence of heterogeneous matrix deposition within tissue samples, suboptimal extraction of analytes, and the occurrence of ion suppression effects (Tobias and Hummon, 2020; Handler et al., 2021). Given the absence of a universally accepted protocol for generating calibration curves and ascertaining tissue concentrations via quantitative mass spectrometry imaging (qMSI), numerous research teams have documented their respective strategies and methodologies for attaining precise quantification and consistent outcomes (Handler et al., 2021).

**FIGURE 7:**
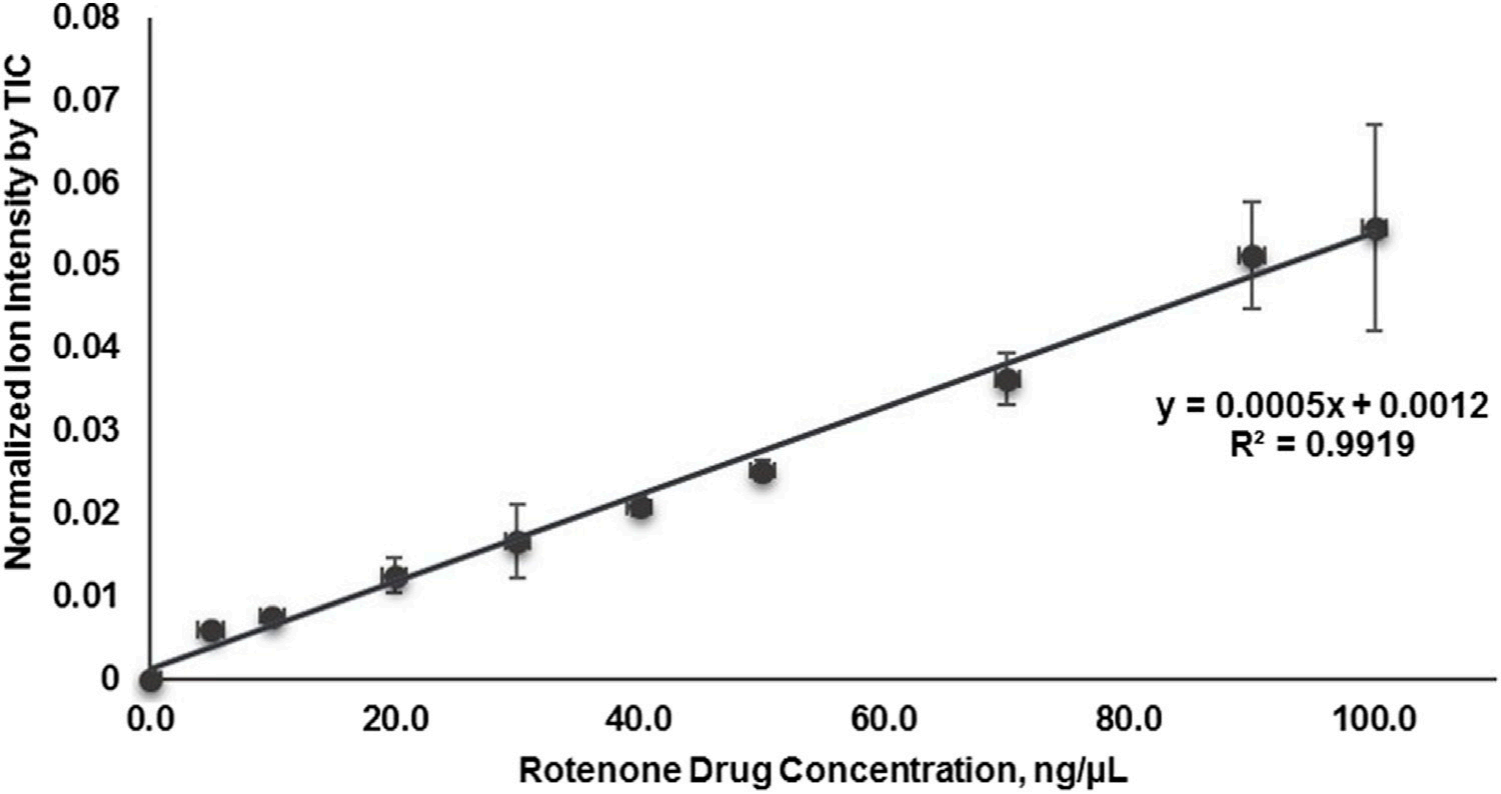
Calibration curve plotted of mean signal intensity ratio of rotenone using MALDI quantitative mass spectrometry imaging (MALDI qMSI).

In this investigation, the relative quantification of the localized distribution of rotenone in the cortical region of kidney tissues was performed using on-tissue qMSI (Supplementary Figure S9). A standard curve was established for rotenone by plotting the mean ion intensities against the nine calibrant concentrations of the rotenone reference drug that were applied to an untreated kidney tissue section (Figure 7; Supplementary Table S2). Based on the calibration curve, the LOD and LOQ were determined as 2.27 × 10^−3^ pg/ng and 6.9210 × 10^−3^ pg/ng in kidney tissue, respectively, at *R* ^2^ = 0.9919 linearity. The relative rotenone concentration in representative tissue sections from seven identified kidney layers was calculated by linear regression of ion intensity data (Schulz et al., 2019) and is presented in Table 3. In order to ascertain the mass of the drug localized in picograms per nanogram of tissue mass (pg/ng), a manual calculation was further conducted utilizing the density (ng/µL) of kidney tissue sections and the calculated concentration (pg/µL) of the drug. The detected amount of rotenone drug in picograms per nanogram of tissue mass ranged from 1.10 × 10^−3^ to 14.7 × 10^−3^ pg/ng. The on-tissue qMSI analysis revealed detectable concentrations of rotenone in representative kidney tissue sections from layers 4, 5, 6, and 7, indicating the presence of rotenone in these layers. Notably, rotenone concentrations exceeding the LOQ were accurately measured in kidney tissue sections from layers 6 and 7. No rotenone was detected in kidney tissues from layers 1, 2, and 3, highlighting the limitations of the qMSI experiment in detecting lower drug concentrations in these layers. The qMSI experiment utilized in this study partially meets the quantitation standards. The successful detection and quantification of rotenone were achieved at higher concentrations in layers 6 and 7; however, the detection and quantification at lower concentrations in layers 1, 2, and 3 could be unreliable (Table 3). Meanwhile, the rotenone detection and quantification in kidney tissue section from layer 4 and 5 is partially reliable (Table 3). Nonetheless, the qMSI analysis may suggest that the observed rotenone distribution in the renal cortex has low concentrations. Even though relatively low levels of rotenone were found, these findings may indicate the rotenone’s potential nephrotoxicity stemming from drug accumulation when administered repeatedly.

**TABLE 3:** Mass intensity and drug quantity of rotenone in treated kidney tissues.

Sample	Normalized Mass intensity by TIC	Concentration (ng/µL)	Relative concentration per tissue section (pg/ng)
Kidney Tissue 1	0.0030	3.6	1.65 × 10^−3^
Kidney Tissue 2	0.0022	2.0	1.10 × 10^−3^
Kidney Tissue 3	0.0025	2.6	1.91 × 10^−3^
Kidney Tissue 4	0.0048	7.2	5.95 × 10^−3^
Kidney Tissue 5	0.0047	7.0	6.42 × 10^−3^
Kidney Tissue 6	0.0052	8.0	9.21 × 10^−3^
Kidney Tissue 7	0.0076	12.8	14.7 × 10^−3^
AVERAGE ± SD	0.0042 ± 0.0019	6.2 ± 3.8	5.86 × 10^−3^ ± 4.94 × 10^−3^

There are several factors contribute to the variability in LOD and LOQ values in the qMSI process. These include instrumental noise, sample preparation, spotting and dilution techniques, calibration curve fitting, and tissue density variations. Addressing these factors can help improve the reliability and accuracy of qMSI experiments. The observed fluctuations in average ion intensities of rotenone, especially at higher concentrations, highlighted the difficulties in attaining reliable signal reproducibility in quantitative MALDI MSI analysis. This inconsistency can be attributed to several plausible factors. One such factor is the formation of heterogeneous crystal sizes within the region of interest which significantly affects the signal intensities in mass spectra. The differences in crystal size give rise to varying ionization efficiencies, whereby larger crystals yield higher signals and smaller crystals yield lower signals. Conversely, smaller crystals may not desorb as efficiently, leading to weaker signals (Knochenmuss, 2021; Zhu X. et al., 2022). Another aspect is the non-uniform deposition of DHB molecules as matrix leading to clumping or uneven coating. This unevenness may affect the ionization process resulting to inconsistent signal intensities. In addition, it is worth noting that the formation of co-crystals between the DHB and rotenone may not be uniform, resulting to spatial variations in ionization efficiency and signal intensity (Knochenmuss, 2021; Zhu X. et al., 2022). Finally, laser conditions may also influence signal reproducibility. The disparity in the laser spot size and position during data acquisition may cause variations on the irradiated sample areas, which would influence the overall variability in signal intensities (Knochenmuss, 2021). These factors pose significant challenges in attaining consistent and reliable quantitation using MALDI mass spectrometry.

## Conclusion

This investigation provides the inaugural documentation of rotenone accumulation in rat kidney, ascertained through MALDI mass spectrometry imaging, without compromising the structural integrity of the tissue. The utilization of MALDI MSI analysis facilitated the elucidation of the spatially confined dispersion pattern of rotenone within the renal tissues of laboratory rats. The cortical region of representative rat kidney tissues exhibits a relatively similar spatial resolution of rotenone adduct ions. When combined with ion mobility spectrometry, MALDI MSI enables the precise and accurate identification of drug metabolites and endogenous compounds. Further investigations are warranted to explore the relationship between the accumulation of drugs in kidney tissues and the efficacy of fragmentation and quantification of drug metabolites. Biomarkers associated with kidney function were discerned by analyzing the variations in ion intensities between untreated and treated tissues. This discovery holds promise in identifying early indicators and biomarkers of drug-induced damage to the kidneys. The utilization of computational tools predicted the biotransformation products of rotenone. Nevertheless, subsequent to a single oral administration lasting 24 h, the water-soluble metabolites were not observed within the renal tissues. The administration of rotenone in a repeated manner may yield enhanced insights into its enduring impacts on vital organs, including the liver, heart, spleen, and lungs. In essence, MALDI MSI offers valuable insights as a preclinical tool for evaluating the spatial distribution of drugs and endogenous metabolites within biological tissues.

## Data Availability

The raw data supporting the conclusion of this article will be made available by the authors, without undue reservation.
