# Supplementary material for: Visualization of renal rotenone accumulation after oral administration and in situ detection of kidney injury biomarkers via MALDI mass spectrometry imaging
Source: Front Mol Biosci. 2024 Jul 1;11:1366278. doi: 10.3389/fmolb.2024.1366278 (PMC11246995; doi:10.3389/fmolb.2024.1366278)
Supplement: Supplementary file 1 [file DataSheet1.pdf]

Visualization of Renal Rotenone Accumulation after Oral Administration and *In Situ* Detection of Kidney Injury Biomarkers via MALDI Mass Spectrometry Imaging

Chuckcris P. Tenebro[1], Neaven Bon Joy M. Marcial[1], Janine J. Salcepuedes[1], Josie C. Torrecampo[1], Rajelle D. Hernandez[3], John Alfon P. Francisco[3], Kristine Mae G. Infante[4], Veronica J. Belardo[4], Monissa C. Paderes[3], Rita Grace Y. Alvero[4], Jonel P. Saludes[2,5,7], Doralyn S. Dalisay[1,6,7*]

[1]Center for Chemical Biology and Biotechnology, University of San Agustin, Iloilo City 5000, Philippines,
[2]Center for Natural Drug Discovery and Development, University of San Agustin, Iloilo City 5000, Philippines
[3]Institute of Chemistry, University of the Philippines Diliman, Quezon City 1101, Philippines
[4]Pharmalytics Corporation, General Trias City 4107, Cavite, Philippines,
[5]Department of Chemistry, University of San Agustin, Iloilo City 5000, Philippines,
[6]Department of Biology, University of San Agustin, Iloilo City 5000, Philippines,
[7]Balik Scientist Program, Department of Science and Technology - Philippine Council for Health Research and Development, Bicutan, Taguig City 1631, Philippines

*Authors to whom correspondence should be addressed: ddalisay@usa.edu.ph

TABLE OF CONTENTS

TABLE S1. Metabolite alteration in kidney tissues dosed with rotenone ………………………..3

TABLE S2. Normalized ion intensities of rotenone standard solutions used in MALDI qMSI….4

FIGURE S1. DriftScope display of the two-dimensional (2D) map of (*m/z*:drift time) of the main fragment ion of rotenone and its isobaric peak …………………………………………....5

FIGURE S2. MALDI MS imaging analysis of possible rotenone detection across treated and untreated kidney tissue sections ....………………………………………………....6

FIGURE S3. MALDI MS imaging analysis of organonitrogen compounds and their corresponding spatial distribution in rotenone-treated kidney tissue sections 7

FIGURE S4. MALDI MS imaging analysis of carboxylic acid and derivatives and their corresponding spatial distribution in rotenone-treated kidney tissue sections 8

FIGURE S5. MALDI MS imaging analysis of purine nucleosides and their corresponding spatial distribution in rotenone-treated kidney tissue sections 9

FIGURE S6. MALDI MS imaging analysis of glycerophospholipids and sphingolipid and their corresponding spatial distribution in rotenone-treated kidney tissue sections 10-11

FIGURE S7. Principal component analysis (PCA) score plot of endogenous metabolites from control and rotenone-treated kidney tissues 12

FIGURE S8. Principal component analysis (PCA) score plot of endogenous biomarkers with significant metabolic alterations in control and rotenone-treated kidney tissues 12

FIGURE S9. Representative MALDI MS images of rotenone drug concentrations spotted on-tissue to generate a calibration standard curve ……………………………………………13

SUPPLEMENTARY INFORMATION

Table S1. Metabolite alteration in kidney tissues dosed with rotenone.

Kidney Metabolite	HMDB ID	Formula	Drift Time (bin)	Adduct Ion	Theoretical *m/z*	Measured *m/z*	Delta ppm	Ion Intensity in Rotenone-treated Kidney Tissues
Choline	HMDB0000097	$C_5H_{13}NO$	18.71	$[M+H]^+$	104.1070	104.1071	0.96	↓***
L-carnitine	HMDB0000062	$C_7H_{15}NO_3$	27.18	$[M+H]^+$	162.1130	162.1125	3.08	↑
Phosphorylcholine	HMDB0001565	$C_5H_{15}NO_4P^+$	29.21	$[M]^+$	184.0733	184.0731	1.09	↑
Adenosine monophosphate	HMDB0011617	$C_{10}H_{14}N_5O_7P$	50.95	$[M+H]^+$	348.0709	348.0707	0.57	↓
Creatinine	HMDB0000562	$C_4H_7N_3O$	19.09	$[M+H]^+$	114.0667	114.0664	2.63	↓
Histidine	HMDB0000177	$C_6H_9N_3O_2$	25.16	$[M+H]^+$	156.0773	156.0770	1.92	↑
Phenylalanine	HMDB0000159	$C_9H_{11}NO_2$	29.45	$[M+H]^+$	166.0859	166.0860	0.60	↓
3-methylhistidine	HMDB0000479	$C_7H_{11}N_3O_2$	27.84	$[M+H]^+$	170.0929	170.0927	1.18	↓
Arginine	HMDB0000517	$C_6H_{14}N_4O_2$	29.03	$[M+H]^+$	175.1195	175.1187	4.57	↓
N-Acetylhistidine	HMDB0032055	$C_8H_{11}N_3O_3$	31.13	$[M+H]^+$	198.0881	198.0881	0.00	↓
O-Phosphotyrosine	HMDB0006049	$C_9H_{12}NO_6P$	40.59	$[M+H]^+$	262.0476	262.0475	0.38	↓
Inosine	HMDB0000195	$C_{10}H_{12}N_4O_5$	49.23	$[M+K]^+$	307.0445	307.0438	2.28	↓**
Adenosine diphosphate	HMDB0000061	$C_{10}H_{15}N_5O_{10}P_2$	60.80	$[M+H]^+$	428.0366	428.0367	0.23	↑
Glycero-3-phosphocholine	HMDB0000086	$C_8H_{20}NO_6P$	45.05	$[M+K]^+$	296.0665	296.0659	2.03	↓*
LysoPC(P-16:0)	HMDB0010407	$C_{24}H_{50}NO_6P$	89.05	$[M+H]^+$	480.3454	480.3431	4.79	↓
LysoPC(16:1)	HMDB0010383	$C_{24}H_{48}NO_7P$	88.47	$[M+H]^+$	494.3230	494.3229	0.20	↑
LysoPC(16:0)	HMDB0010382	$C_{24}H_{50}NO_7P$	91.29	$[M+H]^+$	496.3403	496.3385	3.63	↓
LysoPC(17:0)	HMDB0012108	$C_{25}H_{52}NO_7P$	93.57	$[M+H]^+$	510.3559	510.3539	3.92	↓
LysoPC(18:2)	HMDB0010386	$C_{26}H_{50}NO_7P$	89.73	$[M+H]^+$	520.3403	520.3385	3.46	↑
LysoPC(18:1)	HMDB0010385	$C_{26}H_{52}NO_7P$	93.12	$[M+H]^+$	522.3559	522.3540	3.64	↑
LysoPC(20:4)	HMDB0010395	$C_{28}H_{50}NO_7P$	81.68	$[M+H]^+$	544.3403	544.3378	4.59	↑
LysoPC(18:0)	HMDB0010384	$C_{26}H_{54}NO_7P$	99.13	$[M+K]^+$	562.3275	562.3250	4.45	↓**
Sphingomyelin (D18:1/16:0)	HMDB0010169	$C_{39}H_{79}N_2O_6P$	128.01	$[M+H]^+$	703.5754	703.5723	4.41	↑*

↑ indicates higher ion intensity level than that of the control group; ↓ indicates lower ion intensity level than that of the control group; ▭ organonitrogen compounds ; ▭ carboxylic acid and derivatives ; ▭ purine nucleosides ; ▭ glycerophospholipids and sphingolipid ; (**p* <0.05 ; ***p* <0.01) ; ****p* <0.001)

Table S2. Normalized ion intensities of rotenone standard solutions used in MALDI qMSI.

Rotenone Drug Concentration (ng/μL)	Normalized Ion Intensities of Rotenone Spotted on Blank Kidney Tissues*			
	**Trial 1 (*n=3*)**	**Trial 2 (*n=3*)**	**Trial 3 (*n=3*)**	$\bar{x} \pm \sigma$
5	0.00545 ± 0.00055	0.00637 ± 0.00175	0.00618 ± 0.00033	0.00600 ± 0.00049
10	0.00756 ± 0.00134	0.00716 ± 0.00125	0.00828 ± 0.00019	0.00766 ± 0.00057
20	0.01040 ± 0.00134	0.01267 ± 0.00156	0.01463 ± 0.00061	0.01256 ± 0.00212
30	0.01229 ± 0.00128	0.01657 ± 0.00493	0.02124 ± 0.00455	0.01670 ± 0.00448
40	0.01977 ± 0.00446	0.02205 ± 0.00224	0.02079 ± 0.00353	0.02087 ± 0.00114
50	0.02516 ± 0.00071	0.02650 ± 0.00537	0.02413 ± 0.00096	0.02526 ± 0.00119
70	0.03360 ± 0.00590	0.03556 ± 0.00600	0.03983 ± 0.00716	0.03633 ± 0.00319
90	0.04615 ± 0.00528	0.04888 ± 0.00362	0.05863 ± 0.00385	0.05863 ± 0.00656
100	0.04882 ± 0.00263	0.04605 ± 0.00460	0.06887 ± 0.00812	0.06887 ± 0.01245

*Data from three independent trials with triplicates per calibrant was summarized as mean ($\bar{x}$) ± standard deviation (σ).

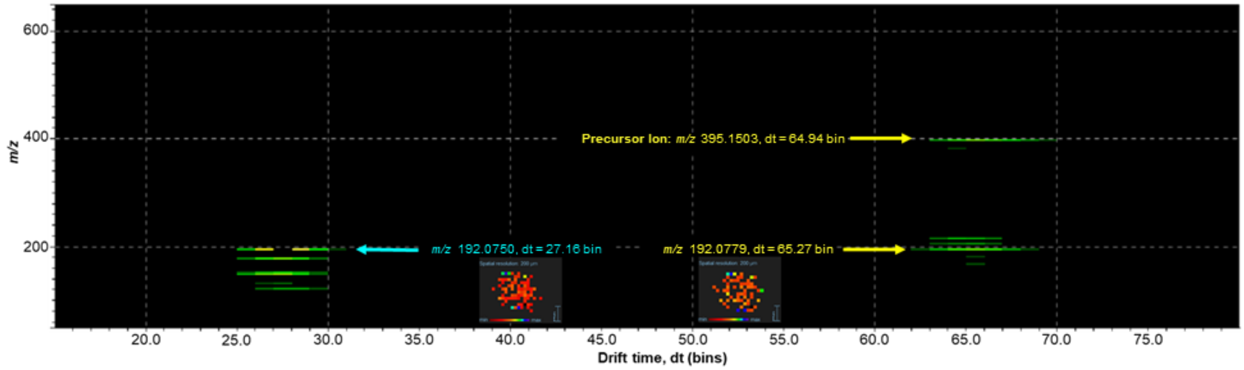

Figure S1. DriftScope display of the two-dimensional (2D) map of (*m/z*:drift time) of the main fragment ion of rotenone and its isobaric peak. At 65.27 bin, the fragment ion (*m/z* 192.0779) aligned with the parent precursor ion (*m/z* 395.1503). A similar peak was detected at *m/z* 192.0750 but has a drift time of 27.16 bin. The MALDI MS images were acquired at 200 µm spatial resolution, and processed using the inverted Weather1 gradient and log scale image composition.

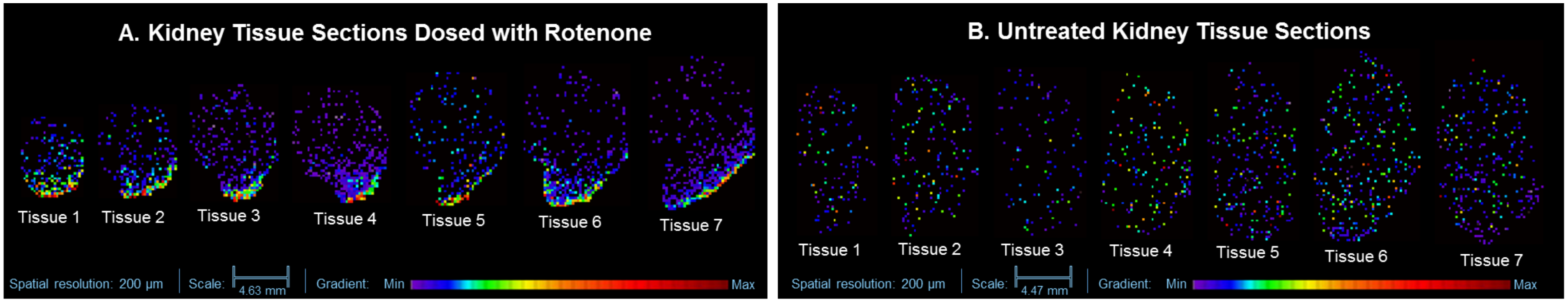

C

Sample	*m/z* of Detected Rotenone 395.1495 [M+H]$^+$	Δ ppm	Ion Intensity	Drift time (bin)	Drift Time Difference (standard - sample)
Treated Kidney Tissues 1 to 7	395.1472 - 395.1484	2.8 - 5.8	228 - 1,033	66.62 - 66.96	0.26 - 0.48
Untreated Kidney Tissues 1 to 7	395.1496 - 395.1587	0.3 - 23.3	73 - 152	63.95 - 71.11	1.23 - 4.63
Rotenone Standard Drug	395.1488 - 395.1492	0.8 - 1.8	164,847 - 183,836	66.36 - 66.48	

Figure S2. MALDI MS imaging analysis of possible rotenone detection across treated and untreated kidney tissue sections. The ion images of protonated rotenone [M+H]$^+$ in (A) drug-treated kidney tissue sections showed a localized distribution in the renal cortex as compared with the (B) untreated kidney tissues. (C) The summary of MALDI MSI analysis demonstrated that detected rotenone in drug-treated kidney tissues has a mass accuracy of less than 6 ppm and a drift time difference of less than 1 bin. These indicators may suggest a plausible rotenone detection in drug-treated kidney tissues.

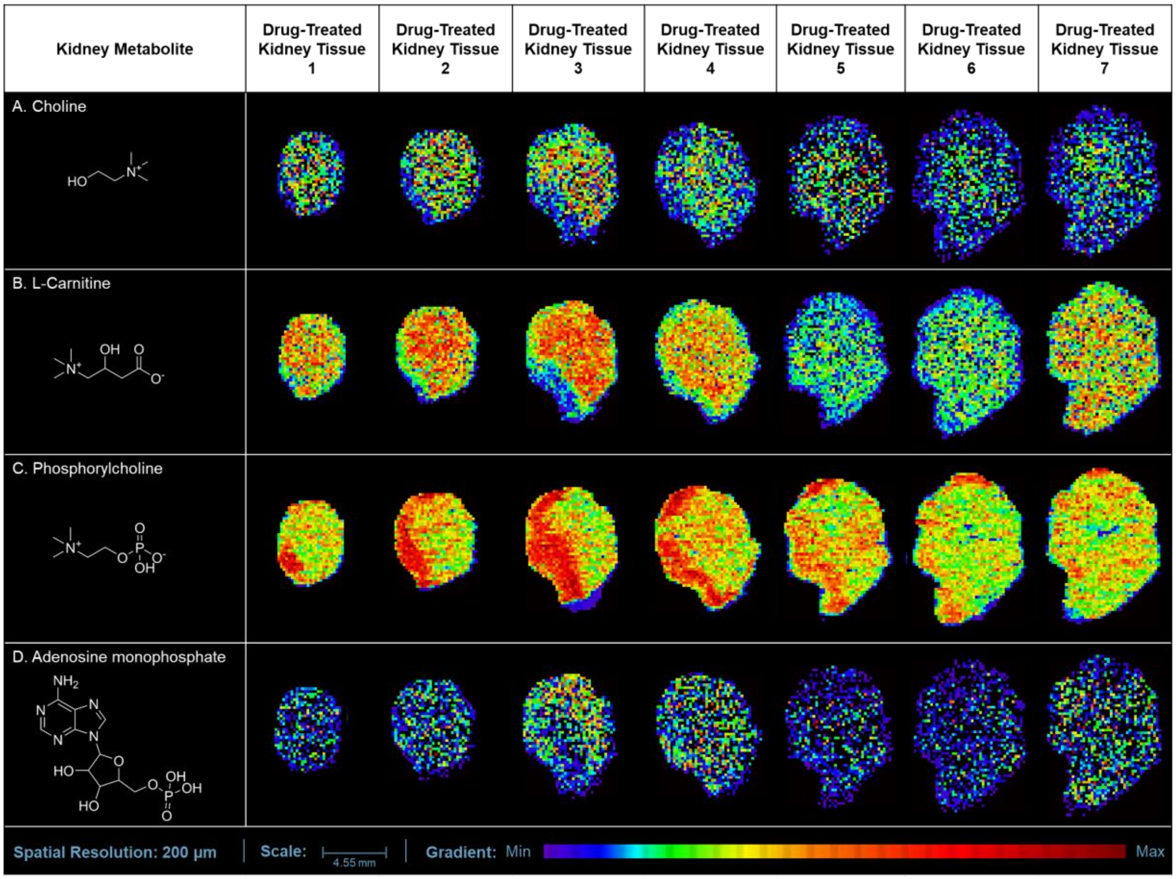

Figure S3. MALDI MS imaging analysis of organonitrogen compounds and their corresponding spatial distribution in rotenone-treated kidney tissue sections. MALDI MS images of (A) choline (*m/z* 104.1071 $[M+H]^+$), (B) L-carnitine (*m/z* 162.1130 $[M+H]^+$), (C) phosphorylcholine (*m/z* 184.0733 $[M]^+$), and (D) adenosine monophosphate (*m/z* 348.0709 $[M+H]^+$) across treated kidney tissue sections (5 µm thickness).

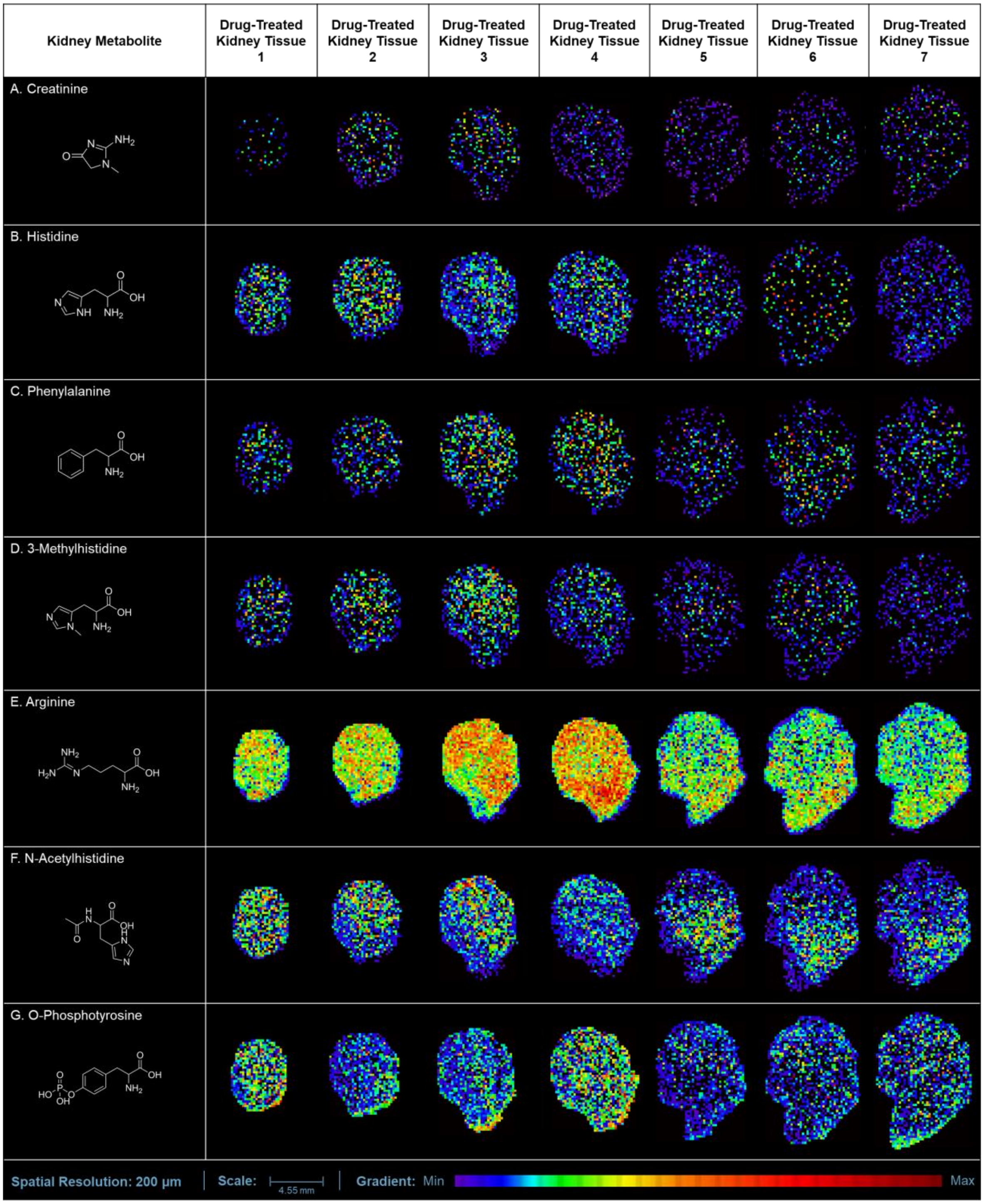

Figure S4. MALDI MS imaging analysis of carboxylic acid and derivatives and their corresponding spatial distribution in rotenone-treated kidney tissue sections. MALDI MS images of (A) creatinine (*m/z* 114.0667 $[M+H]^+$), (B) histidine (*m/z* 156.0773 $[M+H]^+$), (C) phenylalanine (*m/z* 166.0859 $[M+H]^+$), (D) 3-methylhistidine (*m/z* 170.0929 $[M+H]^+$), (E) arginine, (*m/z* 175.1195 $[M+H]^+$) (F) N-acetylhistidine (*m/z* 198.0881 $[M+H]^+$), and (G) O-phosphotyrosine (*m/z* 264.0476 $[M+H]^+$) across treated kidney tissue sections (5 µm thickness).

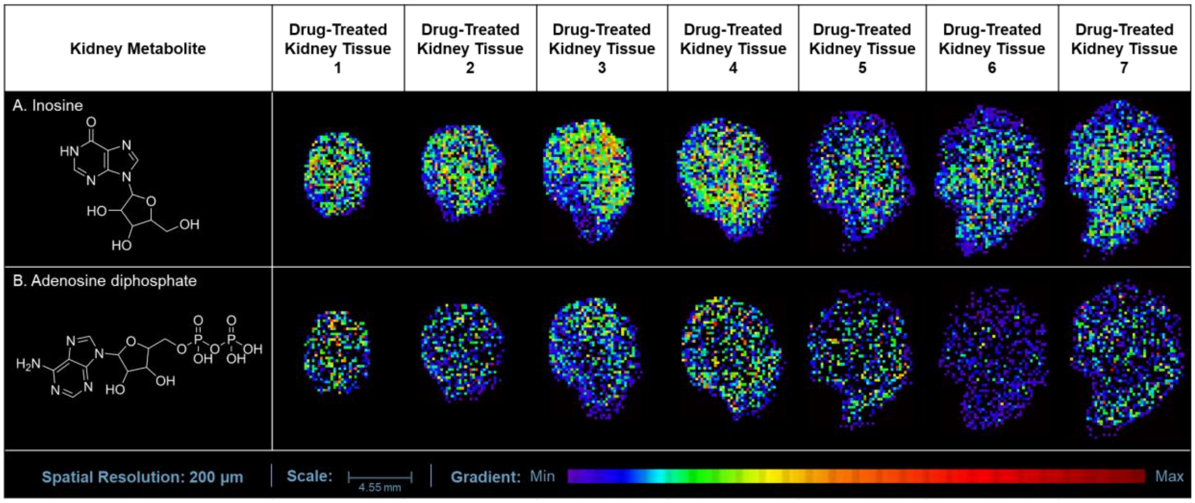

Figure S5. MALDI MS imaging analysis of purine nucleosides and their corresponding spatial distribution in rotenone-treated kidney tissue sections. MALDI MS images of (A) inosine (*m/z* 307.0445 $[M+K]^+$) and (B) adenosine diphosphate (*m/z* 428.0366 $[M+H]^+$) across treated kidney tissue sections (5 µm thickness).

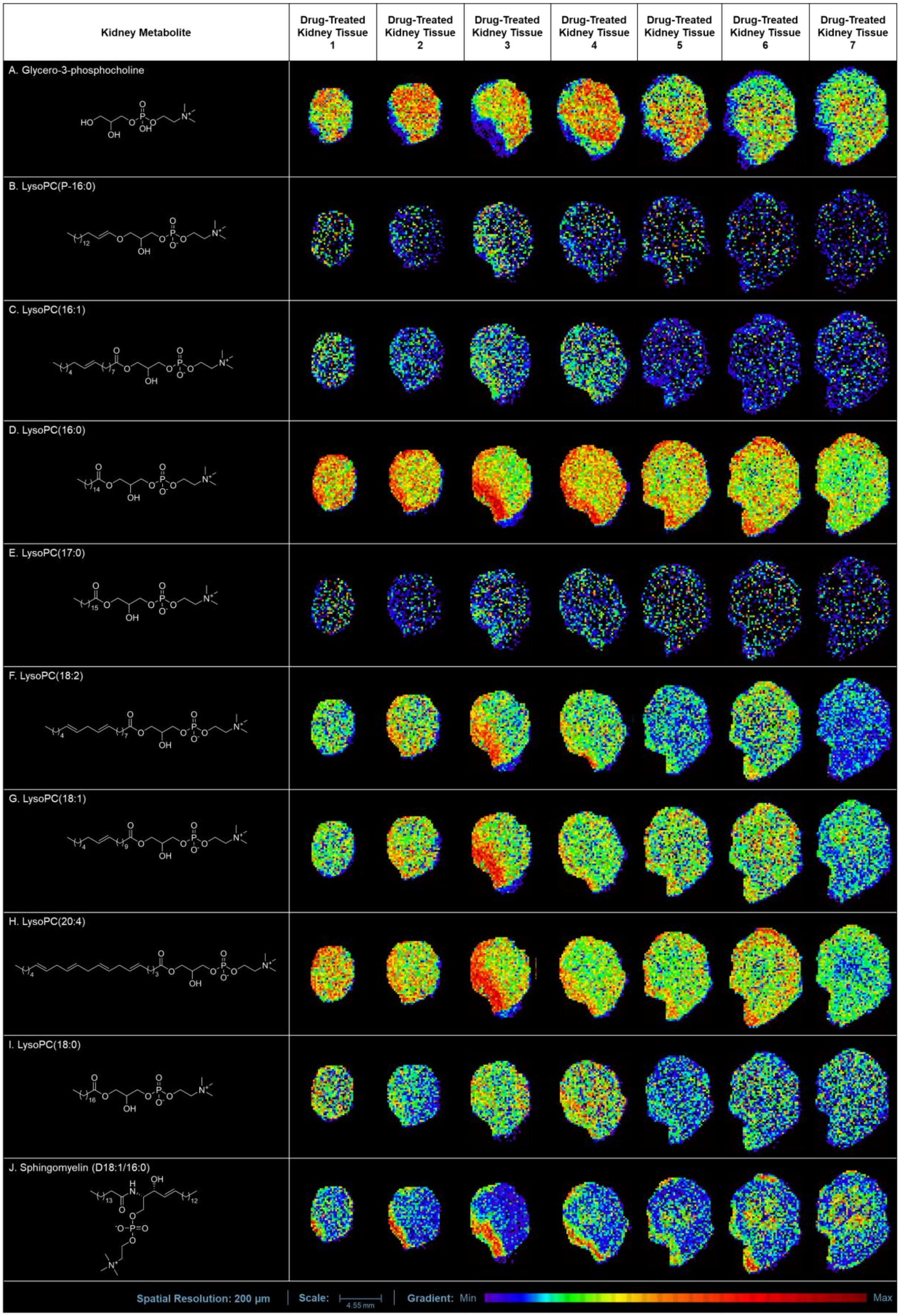
Kidney Metabolite
Drug-Treated Kidney Tissue 1
Drug-Treated Kidney Tissue 2
Drug-Treated Kidney Tissue 3
Drug-Treated Kidney Tissue 4
Drug-Treated Kidney Tissue 5
Drug-Treated Kidney Tissue 6
Drug-Treated Kidney Tissue 7
A. Glycero-3-phosphocholine
B. LysoPC(P-16:0)
C. LysoPC(16:1)
D. LysoPC(16:0)
E. LysoPC(17:0)
F. LysoPC(18:2)
G. LysoPC(18:1)
H. LysoPC(20:4)
I. LysoPC(18:0)
J. Sphingomyelin (D18:1/16:0)
Spatial Resolution: 200 µm
Scale:
4.55 mm
Gradient: Min
Max

Figure S6. MALDI MS imaging analysis of glycerophospholipids and sphingolipid and their corresponding spatial distribution in rotenone-treated kidney tissue sections. MALDI MS images of (A) glycerol-3-phosphocholine (*m/z* 296.0665 $[M+K]^+$), (B) LysoPC(P-16:0) (*m/z* 480.3454 $[M+H]^+$), (C) LysoPC(16:1) (*m/z* 494.3230 $[M+H]^+$), (D) LysoPC(16:0) (*m/z* 496.3403 $[M+H]^+$), (E) LysoPC(17:0) (*m/z* 494.3230 $[M+H]^+$), (F) LysoPC(18:2) (*m/z* 520.3403 $[M+H]^+$), (G) LysoPC(18:1) (*m/z* 510.3559 $[M+H]^+$), (H) LysoPC(20:4) (*m/z* 544.3403 $[M+H]^+$), (I) LysoPC(18:0) (*m/z* 562.3275 $[M+K]^+$), and (J) sphingomyelin (D18:1/16:0) (*m/z* 703.5754 $[M+H]^+$) across treated kidney tissue sections (5 µm thickness).

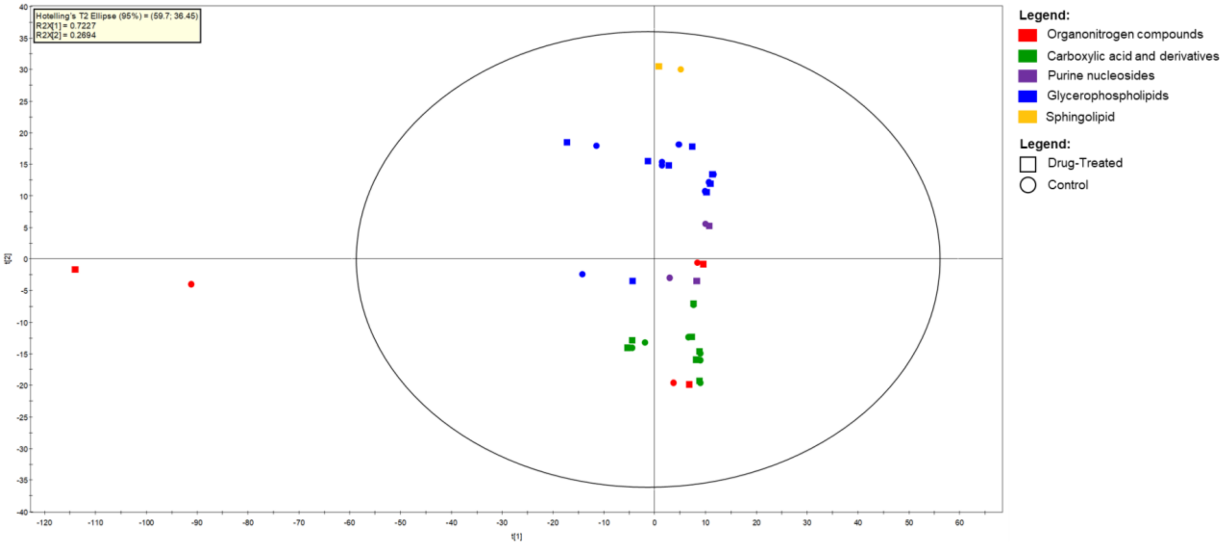

Figure S7. Principal component analysis (PCA) score plot of endogenous metabolites from control and rotenone-treated kidney tissues. The color represented the classes of endogenous compounds, while the shape indicated the experimental groups. Data for each plotted metabolite is the average of the seven cryosections obtained from rat kidneys. The PCA analysis was performed using EZinfo v3.0 (Waters Corporation, Manchester, U.K.).

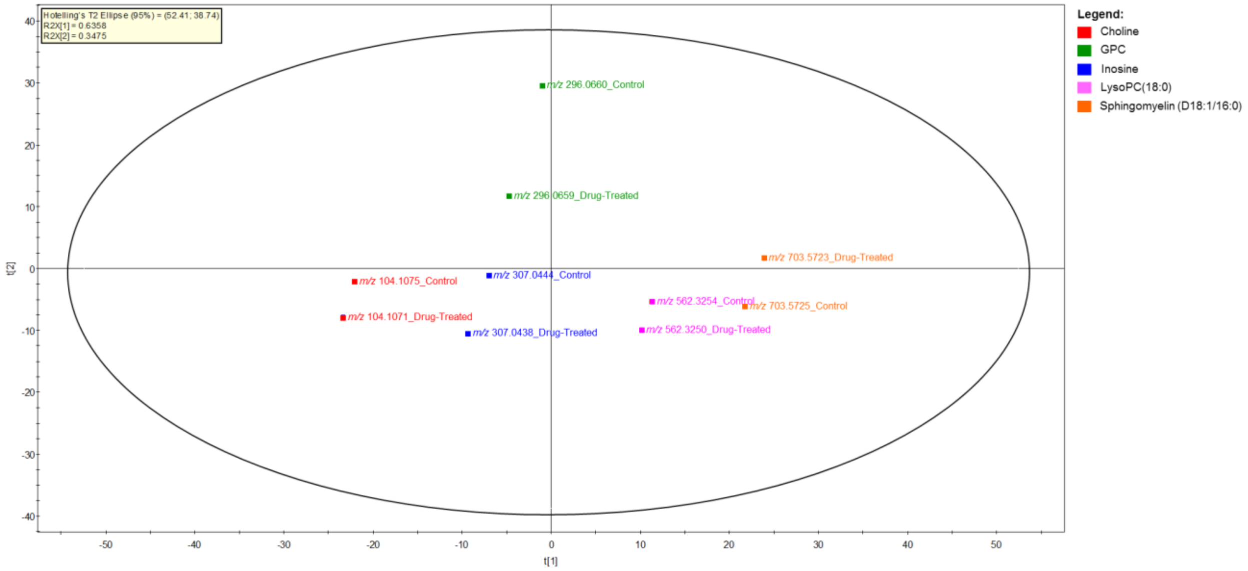

Figure S8. Principal component analysis (PCA) score plot of endogenous biomarkers with significant metabolic alterations in control and rotenone-treated kidney tissues. The color represented the endogenous compounds in the two experimental groups. Data for each plotted metabolite is the average of the seven cryosections obtained from rat kidneys. The PCA analysis was performed using EZinfo v3.0 (Waters Corporation, Manchester, U.K.). (Abbreviation: GPC = glycero-3-phosphocholine; LysoPC = lysophosphatidylcholine).

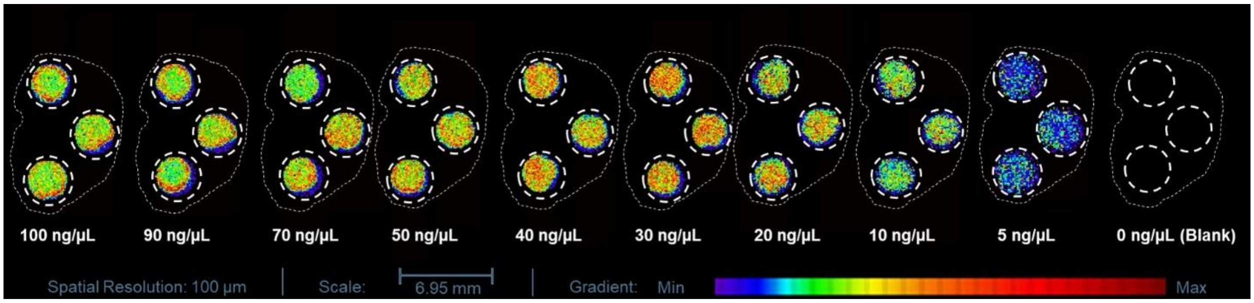

Supporting Information Figure S9. Representative MALDI MS images of rotenone drug concentrations spotted on-tissue to generate a calibration standard curve. Encircled ion images correspond to the detected *m/z* of rotenone standard solutions spotted in three replicates (n=3) on a blank kidney tissue section. MALDI data was obtained at 100 µm spatial resolution and ion images were normalized by their total ion current (TIC).
